# ALDH1A3 is the switch that determines the balance of ALDH^+^ and CD24^−^CD44^+^ cancer stem cells, EMT-MET, and glucose metabolism in breast cancer

**DOI:** 10.1038/s41388-024-03156-4

**Published:** 2024-09-09

**Authors:** Wasundara Fernando, Brianne M. Cruickshank, Raj Pranap Arun, Maya R. MacLean, Hannah F. Cahill, Fiorella Morales-Quintanilla, Cheryl A. Dean, Marie-Claire D. Wasson, Margaret L. Dahn, Krysta M. Coyle, Olivia L. Walker, Melanie R. Power Coombs, Paola Marcato

**Affiliations:** 1 Department of Pathology, Dalhousie University, Halifax, NS Canada; 2 Department of Biology, Acadia University, Wolfville, NS Canada; 3 Department of Surgery, Dalhousie University, Halifax, NS Canada; 4 Department of Biology, Faculty of Science, Saint Mary’s University, Halifax, NS Canada; 5 Department of Molecular Biology & Biochemistry, Simon Fraser University, Burnaby, BC Canada; 6 Department of Microbiology & Immunology, Dalhousie University, Halifax, NS Canada; 7 Nova Scotia Health Authority, Halifax, NS Canada

**Keywords:** Breast cancer, Cancer stem cells

## Abstract

Plasticity is an inherent feature of cancer stem cells (CSCs) and regulates the balance of key processes required at different stages of breast cancer progression, including epithelial-to-mesenchymal transition (EMT) versus mesenchymal-to-epithelial transition (MET), and glycolysis versus oxidative phosphorylation. Understanding the key factors that regulate the switch between these processes could lead to novel therapeutic strategies that limit tumor progression. We found that aldehyde dehydrogenase 1A3 (ALDH1A3) regulates these cancer-promoting processes and the abundance of the two distinct breast CSC populations defined by high ALDH activity and CD24^−^CD44^+^ cell surface expression. While ALDH1A3 increases ALDH^+^ breast cancer cells, it inversely suppresses the CD24^−^CD44^+^ population by retinoic acid signaling-mediated gene expression changes. This switch in CSC populations induced by ALDH1A3 was paired with decreased migration but increased invasion and an intermediate EMT phenotype. We also demonstrate that ALDH1A3 increases oxidative phosphorylation and decreases glycolysis and reactive oxygen species (ROS). The effects of ALDH1A3 reduction were countered with the glycolysis inhibitor 2-deoxy-D-glucose (2DG). In cell culture and tumor xenograft models, 2DG suppresses the increase in the CD24^−^CD44^+^ population and ROS induced by ALDH1A3 knockdown. Combined inhibition of ALDH1A3 and glycolysis best reduces breast tumor growth and tumor-initiating cells, suggesting that the combination of targeting ALDH1A3 and glycolysis has therapeutic potential for limiting CSCs and tumor progression. Together, these findings identify ALDH1A3 as a key regulator of processes required for breast cancer progression and depletion of ALDH1A3 makes breast cancer cells more susceptible to glycolysis inhibition.

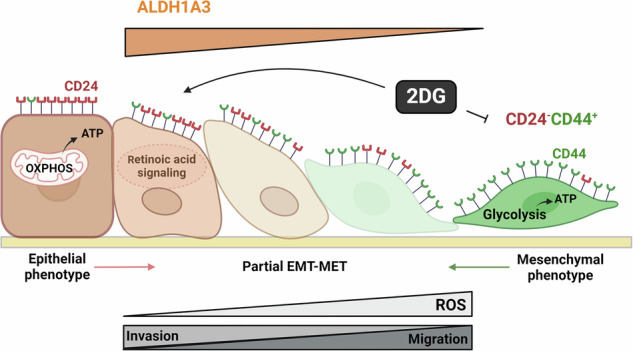

## Introduction

Cancer stem cells (CSCs) are a subpopulation of plastic tumor cells that drive tumor progression and can self-renew and differentiate into cancer cells that make up the bulk of the tumor [1]. CSCs have heightened metabolic plasticity and can exhibit oxidative phosphorylation or glycolysis depending on the conditions of the tumor microenvironment [2]. The plasticity of CSCs is further observed in the switch that occurs in cancer cells for metastasis to occur, where they acquire both epithelial-to-mesenchymal transition (EMT) and mesenchymal-to-epithelial transition (MET) characteristics [3]. The greater resistance of CSCs to chemotherapy and radiation therapy suggests that targeting and limiting CSCs may improve treatment performance and patient prognosis by limiting tumor recurrence and later metastasis [4–6].

For breast cancer, tumor cells that have high aldehyde dehydrogenase (ALDH) activity detected by the Aldefluor assay [7] or the combined low expression of cell surface cluster of differentiation (CD)24 and high expression of CD44 (i.e. CD24^−^CD44^+^ cells) have been defined as CSCs [8]. Although there is evidence of partial overlap between these two CSC populations, with ALDH^+^CD24^−^CD44^+^ breast cancer cells having the greatest tumorigenic potential, the ALDH^+^ and CD24^−^CD44^+^ CSC populations are largely distinct [7]. In fact, the two populations have differing locations within a breast tumor, with ALDH^+^ breast cancer cells more abundant inside the tumor and CD24^−^CD44^+^ cancer cells found more on the periphery [9]. Furthermore, CD24^−^CD44^+^ cancer cells are more mesenchymal and favor glycolytic metabolism, while ALDH^+^ cancer cells are more epithelial and use oxidative phosphorylation [10]. Hence, they are also referred to as epithelial versus mesenchymal breast CSCs. These populations have differing vulnerabilities to stressors typically found in the tumor microenvironment, including low glucose, ROS, and hypoxia [9, 10]. Depending on the conditions of the tumor microenvironment, the plasticity of CSCs allows the cells to transition between the CD24^−^CD44^+^ and ALDH^+^ phenotypes, resulting in continued survival of the tumor [9]. Understanding the factors that govern the transition between these phenotypes will increase our ability to eradicate CSCs and prevent recurrence.

Among the 19 ALDH isoforms, ALDH1A3 and ALDH1A1 are the primary contributors for the high Aldefluor activity that defines ALDH^+^ CSCs of many cancers including, breast, melanoma, glioblastoma, lung, and prostate cancer [11–16]. Focusing on the role of ALDH1A3, we and others, have shown that high ALDH1A3 expression is associated with worse prognosis, promotes tumor progression, invasion, and metastasis, and contributes to chemoresistance in multiple cancers, including melanoma, breast, prostate, glioblastoma and colon cancer [12, 17–30]. In general, these cancer-promoting effects are mediated via ALDH1A3-induced gene expression changes.

For breast cancer, ALDH activity and ALDH1A3 are highest in the aggressive triple-negative breast cancer (TNBC) subtype [29, 31]. Further, compared to other breast cancer subtypes, TNBCs have greater abundances of both ALDH^+^ and CD24^−^CD44^+^ CSC populations. Although inhibiting ALDH1A3 is almost always described as decreasing tumor growth [32–38], in the case of TNBC MDA-MB-468 cells, ALDH1A3 knockdown intriguingly increased the tumor growth; but the metastasis capacity of the cell line decreased upon ALDH1A3 reduction [22]. The mechanisms behind these alternate tumor growth effects of ALDH1A3 in at least MDA-MB-468 cells are unclear; however, considering the recent findings of the switch that can occur between the distinct breast CSC population [10], we wondered if there was a potential compensatory effect. Could the loss of ALDH^+^ CSCs by ALDH1A3 knockdown in the TNBC MDA-MB-468 cells inversely increase the CD24^−^CD44^+^ population, resulting in the cell line retaining its tumorigenic potential?

In this study, we characterize the effect of ALDH1A3 on ALDH^+^ and CD24^−^CD44^+^ populations, EMT and MET, and metabolism in TNBCs. Our results show that while ALDH1A3 increases ALDH^+^ cells, invasion, and oxidative phosphorylation, it inversely decreases CD24^−^CD44^+^ cells, migration, ROS, and aerobic glycolysis. ALDH1A3 reduction rendered the breast cancer cells vulnerable to glycolysis inhibition with inhibitor 2-deoxy-D-glucose (2DG), which reduced the increased CD24^−^CD44^+^ population, migration, ROS, glycolysis, tumor growth, and tumor initiation potential. Together, that data suggests that ALDH1A3 is pivotal in the cell signaling axis that determines the relative abundances between the two distinct CSC populations, EMT-MET, and the balance of aerobic glycolysis versus oxidative phosphorylation.

## Results

### ALDH1A3 increases the ALDH^+^ population while decreasing the CD24^−^CD44^+^ by changing CD24 and CD44 transcript levels and inducing retinoic acid signaling

**Fig. 1:**
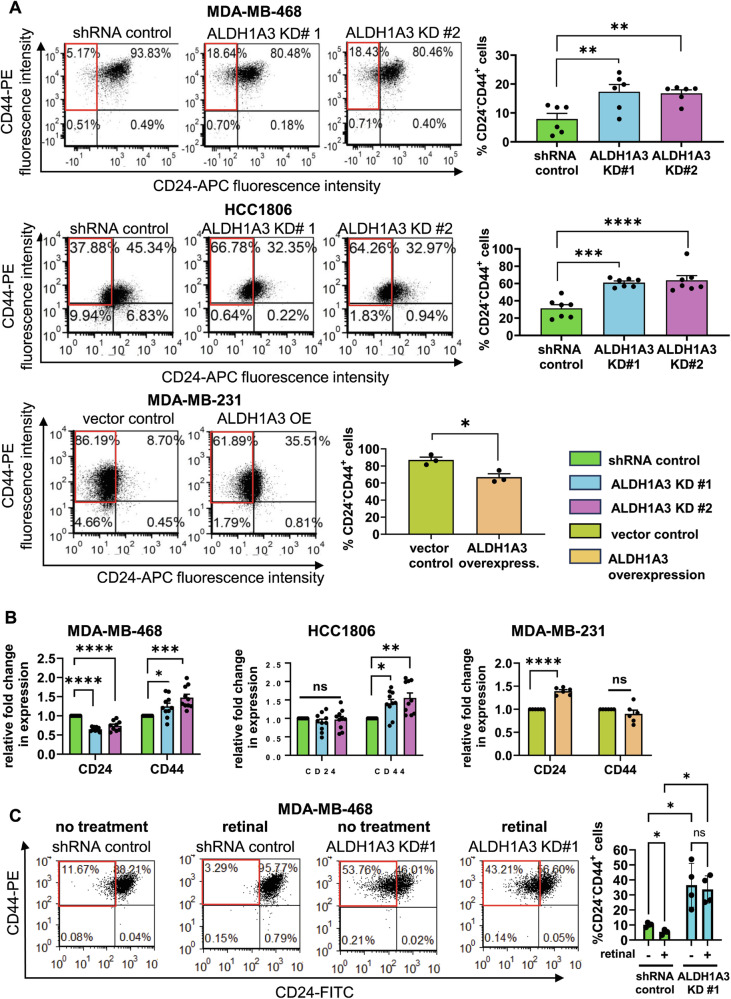
ALDH1A3 and retinal suppress the CD24^−^CD44^+^ cell population in triple-negative breast cancer cells by inducing gene expression changes. **A** The percentage of CD24^−^CD44^+^ cells in MDA-MB-468 and HCC1806 cells, with or without knockdown of ALDH1A3 (by two different shRNA sequences) or in MDA-MB-231 cells with or without ALHD1A3 overexpression is determined by flow cytometry analysis of cell stained with anti-CD24-APC conjugated and anti-CD44-PE conjugated antibody. The bar graphs show the average different biological replicates (6n, MDA-MB-468; 7n HCC1806; and 3n, MDA-MB-231 cells). **B** The effect of ALDH1A3 knockdown or overexpression on the relative mRNA transcript levels of CD24 and CD44 is determined by real-time quantitative polymerase chain reaction (RT-qPCR), relative to two reference genes and the control in MDA-MB-468, HCC1806, and MDA-MB-231 cells (*n* = 8, 8 and 6, respectively). **C** The effect of 24 h 100 nM retinal treatment in MDA-MB-468 cells, with or without ALDH1A3 knockdown, on the percentage of CD24^−^CD44^+^ cells is determined by flow cytometry analysis of cells stained with anti-CD24-FITC conjugated and anti-CD44-PE conjugated antibody (*n* = 4). **A**–**C** The error bars equal standard deviation and significance determined by one-way Anova (**A**, **B**) and two-way Anova in (**C**) and followed by multiple comparison post-tests (*p*-value < 0.05 = *, <0.01 = **, <0.001 = ***, <0.0001 = ****, ns = not significant).

In this study, we use three TNBC cell lines, MDA-MB-468, MDA-MB-231, and HCC1806 cells. MDA-MB-468 and HCC1806 cells have high levels of ALDH1A3 and MDA-MB-231 cells have native low levels of ALDH1A3 (Supplemental Fig. S1, western blots). We therefore knocked down ALDH1A3 in MDA-MB-468 and HCC1806 cells and overexpressed ALDH1A3 in MDA-MB-231 cells to study the effects of the enzyme in the breast cancer cells [29]. We first re-confirmed that high ALDH1A3 increases percentage of ALDH^+^ in the TNBC MDA-MB-468, HCC1806, and MDA-MB-231 cells (Supplemental Fig. S2). Having established the models with altered levels of ALDH1A3 and ALDH activity, we next assessed for potential effects on the percentage of CD24^−^CD44^+^ in the cells upon ALDH1A3 knockdown in MDA-MB-468 and HCC1806 cells, or overexpression ALDH1A3 in MDA-MB-231 cells. This revealed a surprising inverse effect, where high ALDH1A3 inversely results in decreased CD24^−^CD44^+^ cell numbers (Fig. 1A).

Given prior studies demonstrating that effects of ALDH1A3 are mediated via gene expression changes [12, 13, 29], we wondered if the corresponding changes in CD24^−^CD44^+^ cells upon ALDH1A3 knockdown or overexpression originated from changes at the transcript level. RT-qPCR analysis showed that ALDH1A3 knockdown significantly increased the expression of the CD44 in HCC1806 and MDA-MB-468 cells and decreased the expression of CD24 in MDA-MB-468 cells (Fig. 1B). In MDA-MB-231 cells, ALDH1A3 overexpression significantly increased CD24 expression (Fig. 1B). Together, these results suggest that ALDH1A3 suppresses expression of CD44 and/or induces expression of CD24 leading to corresponding changes of CD44 and CD24 on the cell surface of the TNBC cells.

We previously showed that effects on gene expression and tumor growth induced by ALDH1A3 in breast cancer cells were at least partially dependent upon its effects on retinoic acid signaling (i.e. ALDH1A3 converts retinal to retinoic acid, which can then induce gene expression changes through binding hormone receptor ligands) [29, 39]. We therefore treated MDA-MB-468 and HCC1806 cells with the ALDH1A3 substrate retinal for 24 h and then performed the analysis of the CD24^−^CD44^+^ population by flow cytometry. Retinal suppressed the percentage of CD24^−^CD44^+^ population, but only if ALDH1A3 levels were high; knockdown of ALDH1A3 blocked this effect (Fig. 1C, MDA-MB-468 cells; Supplemental Fig. 3S, HCC1806 cells). This is consistent with requiring high levels of ALDH1A3 to convert retinal to the active cell signaling molecule retinoic acid for ALDH1A3 to affect the percentage of CD24^−^CD44^+^ cells. This conclusion is further supported by treating the cells with retinoic acid, which reduced the increased CD24^−^CD44^+^ cells in MDA-MB-468 and HCC1806 cells with ALDH1A3 knockdown (Supplemental Fig. S4). The addition of retinoic acid no longer requires ALDH1A3 enzyme activity to make retinoic acid and induce retinoic acid signaling. Analyses of available chromatin immunoprecipitation with sequencing (ChIP-seq) data [40], provide some evidence of regulation of CD24 and CD44 through the binding of retinoic acid receptor alpha (RARA) within 1 kb of the transcription start of these genes (Supplemental Fig. S5). However, if the effects of ALDH1A3 on CD24 and CD44 gene expression in the TNBC cells is dependent on RAR binding, follow-up ChIP experiments are required with the TNBC cells under conditions where ALDH1A3 and retinoic acid levels are manipulated. It is also likely that ALDH1A3/retinoic acid is altering the expression of the genes independent of RAR binding and retinoic acid response elements (RAREs), which is commonly observed in genes regulated by retinoic acid [39, 41].

We considered the potential impact of the ALDH1A1 enzyme on the observed changes to the CD24^−^CD44^+^ by ALDH1A3, which also has retinaldehyde activity [42]. Previous analyses in MDA-MB-468 cells showed that the knockdown of ALDH1A3 did not impact ALDH1A1 levels and the knockdown of ALDH1A1 in MDA-MB-468 cells did not alter the percentage of ALDH^+^ cells [16, 22]. This suggests that the effects we observe when we manipulate ALDH1A3 levels are likely not due to the indirect effects of ALDH1A1. However, high ALDH1A1 levels in breast cancer cells could similarly affect the CD24^−^CD44^+^ population. Overexpression of ALDH1A1 in MDA-MB-231 cells increased the percentage of ALDH^+^ cells (Supplemental Fig. S6) and reduced the percentage of CD24^−^CD44^+^ cells (Supplemental Fig. S7).

We also assessed how ALHD1A3 levels affect the level of the smaller percentage of hybrid ALDH^+^CD24^−^CD44^+^ cells in the cell lines, which have greater tumor-initiating potential [7] and invasiveness in trans-well assays [9] compared to singly positive ALDH^+^ or CD24^−^CD44^+^ cell populations. In human mammary tissues, these hybrid cells have the greatest mammosphere formation potential and expression of stemness and EMT genes [43]. Knockdown of ALDH1A3 in MDA-MB-468 and HCC1806 cells reduced the percentage of ALDH^+^CD24^−^CD44^+^ cells and in MDA-MB-231 cells, ALDH1A3 overexpression increased the percentage of ALDH^+^CD24^−^CD44^+^ cells (Supplemental Fig. S8), suggesting that ALDH1A3 expression is positively associated with the highly tumorigenic hybrid ALDH^+^CD24^−^CD44^+^ cells. The CD24^+^CD44^+^ population has also been described as having hybrid EMT properties [44]. We noted that upon ALDH1A3 knockdown there was a decrease in the CD24^+^CD44^+^ population and when ALDH1A3 was overexpressed there was an increase in CD24^+^CD44^+^ the population (e.g., 8.7%–35.51%, Fig. 1). We were also curious if ALDH1A3 impacts integrin beta chain 4 (ITGB4) levels in the cells, which has been used to identify TNBC CSCs enriched in mesenchymal cells that have a hybrid EMT phenotype, including MDA-MB-231 cells [45]. Gene expression analysis revealed insignificant changes in MDA-MB-468 and HCC1806 cells but in the mesenchymal MDA-MB-231 cells, ALDH1A3 overexpression reduced the ITGB4 levels (Supplemental Fig. S9). Together this data suggests there are potential associations with ALDH1A3 with hybrid phenotypes and/or its modulation of cells factors associated with hybrid phenotypes within the breast cancer cell lines.

Finally, we wondered if the change in the proportion of ALDH^+^ and CD24^−^CD44^+^ cells can occur due to a change in the proliferation rate of one or both subsets (asymmetric cell division) and/or a switch from one subset to another. Jain et al., provided mathematical modeling that suggests that fluctuations in cellular content duplication and partitioning of EMT transcription factor SNAIL during cell division could explain phenotypic switching and dynamic heterogeneity in PMC42-LA cells [46]. To begin to experimentally address this complex question in our models, we included labeling of HCC1806 cells with the CellTrace^TM^ Violet proliferation kit in flow cytometry analysis, which allows the tracing of multiple generations by dye dilution. This revealed that the CD24^−^CD44^+^ HCC1806 cells have slightly higher division rates than non-CD24^−^CD44^+^ HCC1806 cells and ALDH1A3 knockdown also results in a slightly higher division rate (Supplemental Fig. S10). This suggests that the increased CD24^−^CD44^+^ cells upon reduced ALDH1A3 levels/ALDH^+^ cells are likely partly related to increased cell division of CD24^−^CD44^+^ cells (asymmetric cell division).

### ALDH1A3 induces a partial EMT phenotype in breast cancer associated with gene and protein expression changes

**Fig. 2:**
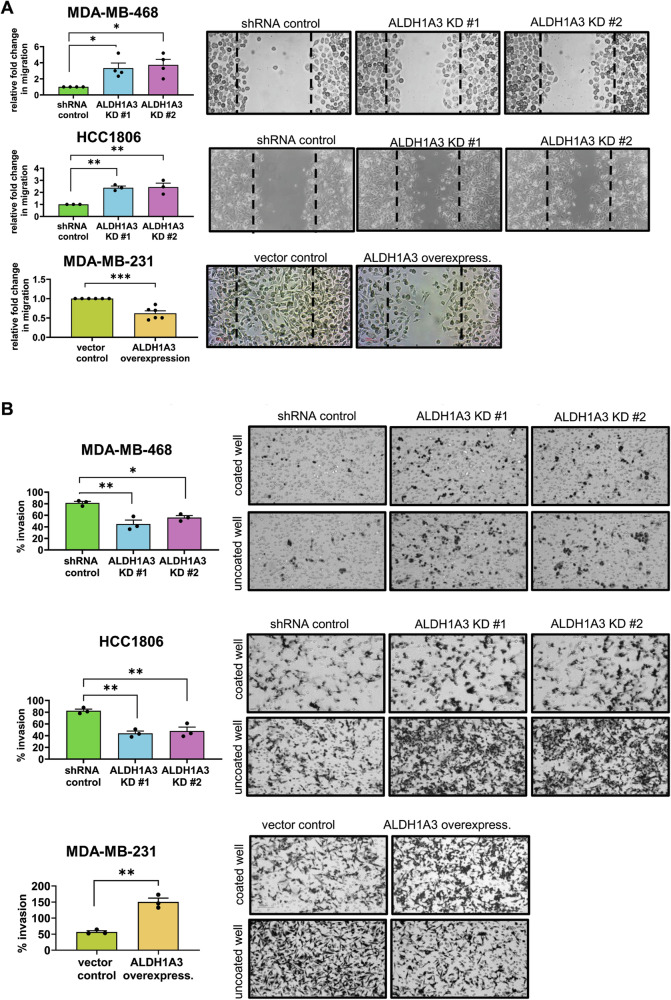
ALDH1A3 inhibits migration but increases invasion in triple-negative breast cancer cell lines. **A** The migration capacity of MDA-MB-468 and HCC1806 cells, with or without knockdown of ALDH1A3 (by two different shRNA sequences) or in MDA-MB-231 cells with or without ALHD1A3 overexpression is determined by gap-closure assays. The images are representative of one of the biological replicates and the bar graphs show the average different biological replicates (4n, MDA-MB-468; 3n, HCC1806; and 6n, MDA-MB-231 cells). **B** The invasive capacity of MDA-MB-468 and HCC1806 cells, with or without knockdown of ALDH1A3 (by two different shRNA sequences) or in MDA-MB-231 cells with or without ALHD1A3 overexpression is determined by trans-well invasion assay where the % of migrated cells in the uncoated well is divided the % of migrated cells in the coated well for each biological replicate and made relative to the control cells. The images are representative of one of the biological replicates and the bar graphs show the average different biological replicates (3n). **A**, **B** The error bars equal standard deviation and significance determined by one-way ANOVA, followed by multiple comparison post-tests (*p*-value < 0.05 = *, <0.01 = **, <0.001 = ***, ns = not significant).

We next wondered if the increase in CD24^−^CD44^+^ cells upon reduced ALDH1A3 levels in the TNBC cells also resulted in phenotypic changes associated with the CD24^−^CD44^+^ CSC population. CD24^−^CD44^+^ cells are characteristically mesenchymal and have increased expression of mesenchymal markers [9]. ALDH1A3 knockdown in MDA-MB-468 and HCC1806 cells resulted in increased migration in the gap-closure assay (Fig. 2A). Furthermore, overexpression of ALDH1A3 decreased the migration of MDA-MB-231 cells in the gap-closure assay (Fig. 2A) suggesting restoration of epithelial characteristics in the mesenchymal MDA-MB-231 cells [9, 47–49]. However, the invasion relative to the migration was higher in ALDH1A3-expressing cells in trans-well assays, especially in MDA-MB-231 cells (Fig. 2B). Invasion is a feature attributed to mesenchymal cells that demonstrate an EMT phenotype [50]. Therefore, the mixed phenotype of decreased migration (Fig. 2A)/increased invasion (Fig. 2B) imparted by ALDH1A3 suggests that ALDH1A3 promotes an intermediate or partial EMT phenotype that has been ascribed to CSCs [51].

**Fig. 3:**
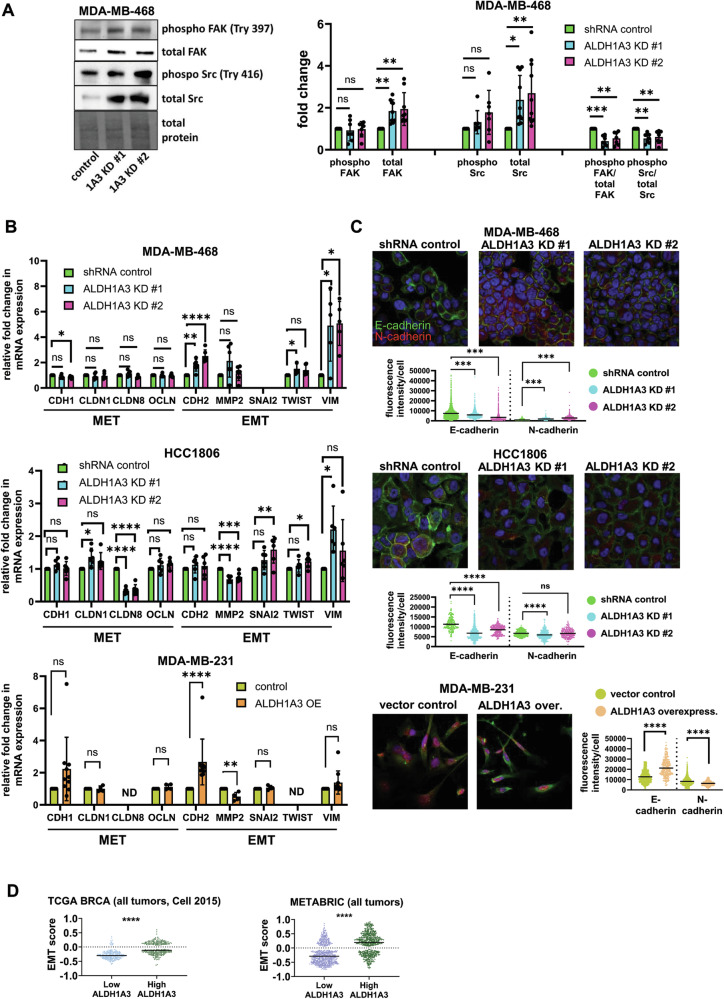
ALDH1A3 increases alters FAK and Src levels and ratios of phosphorylated FAK and Src, increases E-cadherin and decreases N-cadherin by immunofluorescence, and alters expression of MET and EMT genes and the EMT score. **A** The effect of ALDH1A3 knockdown or overexpression on the protein levels of phosphorylated (Tyr 397) and total focal adhesion kinase (FAK) and phosphorylated (Try 416) and total Src in MDA-MB-468 cells quantified by western blotting (7n with phospho antibodies, 9n for total FAK antibody, and 10n for total Src antibody). The bar graphs summarize the image band quantification of individual biological replicates relative to the total protein and the control and the ratio of phosphorylated FAK or Src versus total FAK or Src for 7 complete sets. **B** The effect of ALDH1A3 knockdown or overexpression on the relative mRNA transcript levels of mesenchymal-epithelial-transition (MET) genes CDH1, CLDN1, CLDN2, OCLN, and epithelial-mesenchymal-transition (EMT) genes CDH2, MMP2, SNAI2, TWIST, and VIM is determined by quantitative polymerase chain reaction (RT-qPCR), relative to two reference genes and the control in MDA-MB-468 (6n), HCC1806 (6n), and MDA-MB-231 cells (4–10n). **C** The effect of ALDH1A3 knockdown or overexpression on E-cadherin and N-cadherin in MDA-MB-468, HCC1806, and MDA-MB-231 cells is visualized in immunofluorescence images and fluorescence intensity of the protein staining in >200 cells per conditions is quantified. The line in the dot plots indicates the mean. **A**–**C** Significance determined by one-way ANOVA followed by multiple comparison post-tests and *p*-value < 0.05 = *, <0.01 = **, <0.001 = ***, <0.0001 = ****, ns = not significant. Error bars represent standard deviation. **D** The EMT score is calculated for individual patient tumors using expression data available for TCGA BRCA (Cell 2015) and METABRIC datasets. Patient tumors are grouped as either low or high ALDH1A3 based on the ranking of being in the bottom third for ALDH1A expression or the top third of all breast cancer patients within the dataset. The line represents the mean. Significance determined by unpaired *t*-test.

We next performed western blotting, gene expression, and microscopy analyses to investigate for potential molecular changes induced by ALDH1A3 in the breast cancer cells that would result in the intermediate/partial EMT phenotype we observed in Fig. 2 (Fig. 3). During cell motility, the cell membrane is deformed by depolymerization of the actin cytoskeleton so that the focal adhesion proteins between the cell and extracellular matrix move towards the leading edge [52]. This is initiated by the FAK and Src complex, two non-receptor tyrosine kinases, which mediate the cell signaling critical in regulating cell migration and invasion processes. In breast cancer cells, invasion was dependent upon upstream phosphorylation of FAK tyrosine 397 and subsequent phosphorylation of Src [53]. We assessed the levels of FAK and Src in the cells by western blotting and noted that ALDH1A3 knockdown in MDA-MB-468 cells and HCC1806 cells increases total FAK and Src levels, while overexpression of ALDH1A3 in MDA-MB-231 cells decreases total FAK and Src levels (Fig. 3A, MDA-MB-468 cells; Supplemental Fig. 11, HCC1806 and MDA-MB-231 cells). Interestingly, the levels of phosphorylation of these proteins in general were not concomitantly increased alongside the increased total protein levels upon reduced ALDH1A3 (Fig. 3A, MDA-MB-468 cells; Supplemental Fig. 11, HCC1806 and MDA-MB-231 cells). This results in a significant reduction in the ratio of phosphorylated FAK or Src versus total FAK or Src upon reduction of ALDH1A3 (Fig. 3A, MDA-MB-468 cells; Supplemental Fig. 11, HCC1806 and MDA-MB-231 cells). Given, the importance of FAK and Src in cell motility and invasion [53–55], the hybrid phenotype of increased total FAK and Src, but impaired phosphorylation of the increased proteins, upon ALDH1A3 reduction is likely contributing to hybrid phenotype of increased migration/decreased invasion (Fig. 2).

We investigated the gene expression changes in the cell lines by performing RT-qPCR on well-known EMT/MET markers [9, 43, 48–50]. In MDA-MB-468 cells, ALDH1A3 knockdown decreased expression of epithelial marker CDH1, which encodes the cell junction E-cadherin protein, and increased the expression of mesenchymal markers CDH2, TWIST1 and VIM, which encode N-cadherin, twist and vimentin, respectively (Fig. 3B). Overall, ALDH1A3 knockdown in MDA-MB-468 cells had decreased expression of EMT markers and increases expression of MET markers (Fig. 3B). In HCC1806 cells ALDH1A3 knockdown had a mixed effect on expression of EMT/MET marker genes. ALDH1A3 knockdown decreases expression of epithelial marker CLDN8 (encodes claudin 8) and increases expression of some mesenchymal markers (SNAI2 (encodes slug), TWIST1, and VIM, Fig. 3B); however, we also noted opposing effects with increased expression of epithelial marker CLDN1 (encodes claudin 1) and decreased expression of mesenchymal marker MMP2 (encodes matrix metalloproteinase 2). In MDA-MB-231 cells there was a significant increase in the expression of mesenchymal marker CDH2 (Fig. 3B). Thus, in the TNBC cell lines, we observe that ALDH1A3 induces expression changes in both EMT and MET markers, but not uniformly in one direction.

We next assessed if the gene expression changes in CDH1 and CDH2 induced by ALDH1A3 in the breast cancer cells translate to protein changes in the cells. We visualized the adherens junction proteins E-cadherin and N-cadherin in cell cultures by immunofluorescence microscopy of cells grown on coverslips. ALDH1A3 knockdown decreases E-cadherin in MDA-MB-468 and HCC1806 cells, which is predominately membrane-localized (Fig. 3C). In contrast, N-cadherin is more diffuse throughout the cells and is increased by knockdown. Consistently, ALDH1A3 overexpression increases E-cadherin and decreases N-cadherin in MDA-MB-231 cells (Fig. 3C). We noted visible changes in the appearance of the HCC1806 cells upon ALDH1A3 knockdown, (Fig. 3C), with altered cell junctions compared to control cells. Overall, the increase in E-cadherin/decrease in N-cadherin in cells with low ALDH1A3 is consistent with the increase in migration observed in Fig. 2A.

We next investigated how ALDH1A3 affects the overall EMT score of breast cancer patient tumors. The EMT score is calculated using the Kolmogorov–Smirnov metric, which quantifies the difference between the empirical cumulative distributive functions for the epithelial versus the mesenchymal gene signatures, consisting of over 300 genes [56]. The EMT score ranges between −1 to +1, with positive scores indicative of a more mesenchymal phenotype and negative scores indicative of a more epithelial phenotype, and a neutral score would equate to a hybrid EMT-MET phenotype. We calculated the EMT score of breast cancer patient tumors from TCGA BRCA and METABRIC datasets grouped as having low ALDH1A3 levels (bottom third of patient tumors) versus high ALDH1A3 (top third of patient tumors). In the TCGA BRCA and METABRIC breast cancer patient tumor datasets, patient tumors with high ALDH1A3 had higher EMT scores, with mean EMT scores closer to neutral (Fig. 3D). Separating by subtype, we noticed the same trends of ALDH1A3 levels correlations with EMT scores in ER^+^, HER2^+^, and TNBC, especially in ER^+^ breast cancers (Supplemental Fig. S12). Interestingly, the association of ALDH1A3 with EMT score is less prominent and not significant in TNBC. Although perhaps unexpected, this could be due to TNBCs having higher EMT scores overall and relatively fewer patient numbers. It also suggests that the effects of ALDH1A3 in the other breast cancer subtypes should be investigated. To be more meaningful, the EMT score analysis should be conducted on expression data from single cells, which would allow for the separate analysis of ALDH1A3^+^ versus ALDH1A3^–^ cells within the same tumor. Together, the results in Figs. 2 and 3 suggest that ALDH1A3 promotes an intermediate/partial EMT phenotype resulting in decreased migration and increased invasion, by inducing gene expression and protein changes in breast cancer associated with both EMT and MET phenotypes.

### ALDH1A3 shifts glucose metabolism towards decreased glycolysis and increased oxidative phosphorylation, especially in MDA-MB-468 cells

**Fig. 4:**
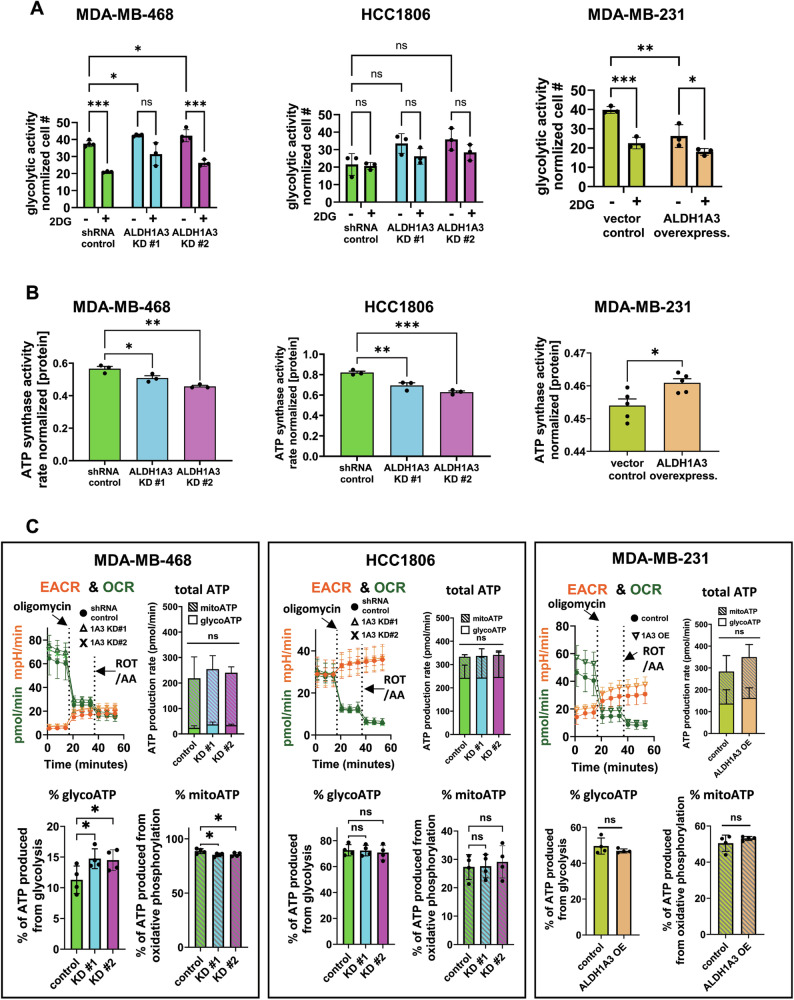
ALDH1A3 decreases glycolytic activity, increases ATP synthase activity, and in MDA-MB-468 cells suppresses ATP production from glycoATP and increases mitoATP. **A** The glycolytic activity of MDA-MB-468 and HCC1806 cells, with or without knockdown of ALDH1A3 (by two different shRNA sequences) or in MDA-MB-231 cells, with or without ALHD1A3 overexpression is determined by a fluorescent Glycolysis Assay with a plate reader (*n* = 3). As a control, treatment of the cells 5 mM of the the glycolysis inhibitor 2-deoxy-D-glucose (2DG) is included. **B** The ATP synthase activity of lysates of MDA-MB-468 and HCC1806 cells, with or without knockdown of ALDH1A3 (by two different shRNA sequences, *n* = 3) or in MDA-MB-231 cells with or without ALHD1A3 overexpression is determined by microplate assays (*n* = 5). **C** The live cell Seahorse ATP rate assay is conducted for MDA-MB-468 and HCC1806 cells, with or without knockdown of ALDH1A3 (by two different shRNA sequences) or in MDA-MB-231 cells with or without ALHD1A3 overexpression. The EACR and OCR plots are shown, along with the total ATP production rate and relative ATP production rate from glycoATP (glycolysis) and mitoATP (oxidative phosphorylation) bar graphs (*n* = 4). **A**–**C** The error bars equal standard deviation and significance determined by two-way ANOVA in (**A**) and one-way ANOVA in (**B**, **C**) for MDA-MB-468 and HCC1806 cells, followed by multiple comparison post-tests. For MDA-MB-231 cells in (**B**, **C**), we performed *t*-tests. Significance is indicated as follows: *p*-value < 0.05 = *, <0.01 = **, <0.001 = ***, ns = not significant.

Given that CD24^−^CD44^+^ cells also have enhanced aerobic glycolytic activity [10], and ALDH1A3 decreases the CD24^−^CD44^+^ population (Fig. 1), we hypothesized that ALDH1A3 affects the glucose metabolism of the breast cancer cells. We performed a glycolysis assay (extracellular acidification) which measures lactate production and the glycolytic flux of the breast cancer cells. As a control, we treated the cells with 2DG, the glycolysis inhibitor, which reduces glycolysis in MDA-MB-468 and MDA-MB-231 cells (an insignificant trend in HCC1806 cells, Fig. 4A). Knockdown of ALDH1A3 increases glycolysis (extracellular acidification - lactate production) in MDA-MB-468, and overexpression of ALDH1A3 decreases the glycolysis/lactate production MDA-MB-231 cells (Fig. 4A). The effect was not significant in HCC1806 cells.

We performed an ATP synthase assay in the cells, which measures the activity of ATP synthase. ATP synthase catalyzes the production of ATP and is Complex V of the electron transport chain, the final step of oxidative phosphorylation. ALDH1A3 knockdown in MDA-MB-468 and HCC1806 cells decreases ATP synthase activity and ALDH1A3 overexpression in MDA-MB-231 cells increases ATP synthase activity (Fig. 4B). Together with the inverse effects on extracellular acidification (Fig. 4A), the increased ATP synthase activity imparted by ALDH1A3 (Fig. 4B), suggests that high ALDH1A3 could be shifting the metabolism of the cells towards more active oxidative phosphorylation.

We performed the gold standard assay for assessing ATP production by glycolysis and oxidative phosphorylation by completing the Seahorse XF ATP Real-Time rate assay. The assay simultaneously measures extracellular acidification rates (ECAR) and oxygen consumption rates (OCR) and can quantify the relative balance between oxidative phosphorylation and glycolysis and the rate of ATP production from glycolytic and mitochondrial systems using label-free technology in live cells (Fig. 4C). This revealed that MDA-MB-468 cells generate most of their ATP through oxidative phosphorylation, with very little ATP generated from glycolysis and ALDH1A3 knockdown increased ATP production from glycolysis (Fig. 4C, left). In contrast, HCC1806 generated most of the ATP though glycolysis and ALDH1A3 knockdown did not alter the dynamics of ATP production. MDA-MB-231 cells generate ATP evenly through both glycolysis and oxidative phosphorylation and ALDH1A3 did not significantly alter this ratio in the cells. This data suggests that ALDH1A3 knockdown in MDA-MB-468 cells could provide a tumor growth advantage for this cell line [29] since the loss of ALDH1A3 makes the cells generate more ATP from aerobic glycolysis, which is required for tumor growth and progression in vivo [57–62] and aerobic glycolysis is minimal in the cell line (Fig. 4C).

**Fig. 5:**
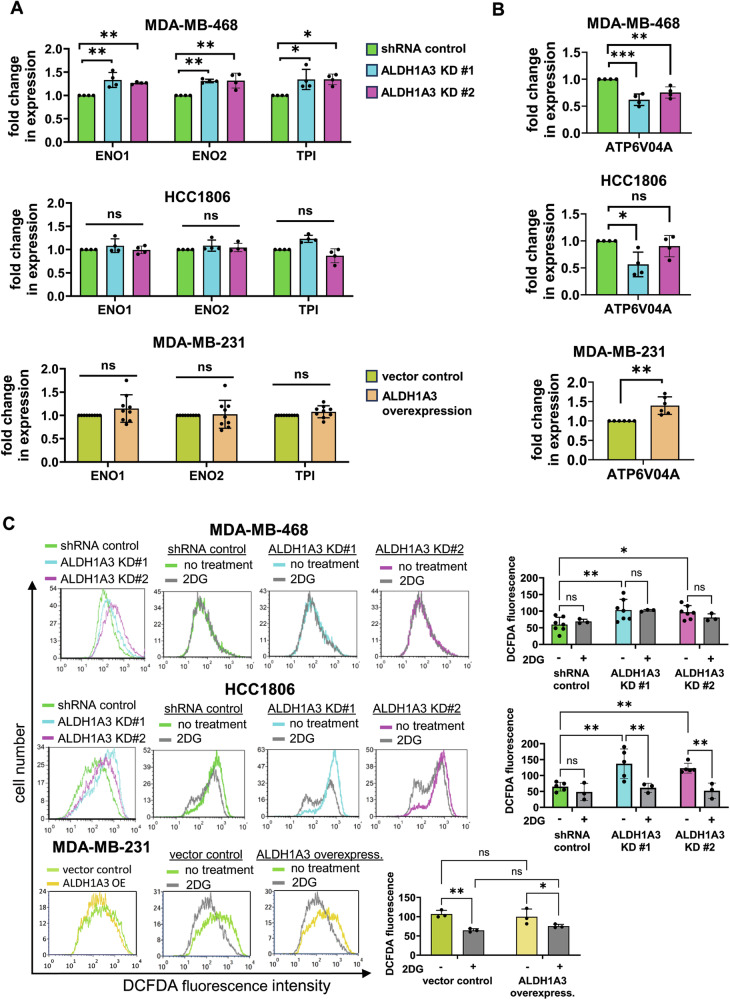
ALDH1A3 suppresses expression of glycolysis genes in MDA-MB-468 cells and increases expression of ATP synthase gene and ROS levels in triple-negative breast cancer cells. **A**, **B** The effect of ALDH1A3 knockdown or overexpression on the relative mRNA transcript levels of ENO1, ENO2, and TPI (**A**) and ATP6V04A (**B**) is determined by real-time quantitative polymerase chain reaction (RT-qPCR), relative to two reference genes and the control in MDA-MB-468 (*n* = 4), HCC1806 (*n* = 4), and MDA-MB-231 (*n* = 9 in A, *n* = 6 in **B**) cells. **C** The effect of ALDH1A3 knockdown or overexpression or 48 h 5 mM 2-deoxy-D-glucose (2DG, *n* = 3) on reactive oxygen species (ROS) is quantified by flow cytometry analysis of dichlorodihydrofluorescein diacetate (H_2_DCFDA) stained cells (*n* = 7 for MDA-MB-468 cells, *n* = 5 for HCC1806 cells, and *n* = 3 for MDA-MB-231 cells). Representative experiments are shown for each cell line and the bar graph summarizes the results of individual biological replicates. **A**–**C** The error bars equal standard deviation and significance determined by one-way ANOVA (or *t*-test for MDA-MB-231 cells) in (**A**, **B**) and two-way ANOVA in (**C**), followed by multiple comparison post-tests (*p*-value < 0.05 = *, <0.01 = **, <0.001 = ***, ns not significant).

We investigated potential mechanisms of the effects of ALDH1A3 on glucose metabolism by evaluating gene expression changes of key genes that mediate glycolysis and oxidative phosphorylation and ROS levels (since ROS is a major cellular inducer of aerobic glycolysis [63–65]). This revealed that ALDH1A3 knockdown in MDA-MB-468 cells increased expression of the key glycolysis enzymes enolase 1 and 2 (ENO1 and ENO2), and triosephosphate isomerase (TPI) [66–68] (Fig. 5A). In HCC1806 and MDA-MB-231 cells, the expression of the genes was not altered by ALDH1A3. We also evaluated for effects on gene expression of key oxidative phosphorylation genes of the electron transport chain. This revealed consistent effects of ALDH1A3 on the expression of ATPase H^+^ transporting V0 subunit a4 (ATP6V04A) (Fig. 5B). These gene expression changes could explain the shift of glucose metabolism we observed in MDA-MB-468 cells (Fig. 4C), and the overall increased ATP synthase activity imparted by ALDH1A3 in the cell lines (Fig. 4B). We also considered the possibility that ALDH1A3 was affecting glycolysis indirectly through expression effects on master regulator of glycolytic metabolism, nuclear factor erythroid 2‐related factor 2 (NRF2) [69, 70], which was also shown to be modulator of the hybrid EMT/MET phenotype in cancer cells [10, 71]. Although we failed to note changes in expression in NRF2 mRNA levels (Supplemental Fig. S13), it does not negate the possibility that the activity of the transcription factor is altered, and inhibition of the protein may reveal the impact of NRF2 on the ALDH1A3-mediated effects.

We measured ROS levels in the cells by staining the cells with H_2_DCFDA and noted a significant increase in ROS when ALDH1A3 was knocked down in MDA-MB-468 and HCC1806 cells (Fig. 5C), which could be dampened by the addition of ROS-scavenger NAC (Supplemental Fig. S14). Overexpression of ALDH1A3 in MDA-MB-231 cells did not induce a significant change in ROS. ALDH enzymes oxidize ROS-generating aldehydes in cells [72]; therefore, the increased ROS in ALDH1A3 knockdown cells is likely related to this oxidation activity. Previous reports suggest ROS can be inhibited or increased by 2DG [73, 74]. In breast cancer MCF7 cells and murine mammary 4T1 cells, 2DG inhibition of glycolysis occurs through ROS inhibition and a cell signaling feedback loop (2DG/ROS/PI3K/AKT/HIF1α/HK2/glycolysis) [73]. Treatment of the TNBC cells with 2DG inhibits ROS overall and the ROS increased upon ALDH1A3 knockdown (Fig. 5C). Together, this data suggests that the shift toward aerobic glycolysis induced upon ALDH1A3 reduction, especially in MDA-MB-468 cells (Fig. 4) could be connected to the increased ROS and gene expression changes observed upon ALDH1A3 reduction.

### 2DG inhibits the increased migration and increased CD24^−^CD44^+^ population induced by ALDH1A3 knockdown

**Fig. 6:**
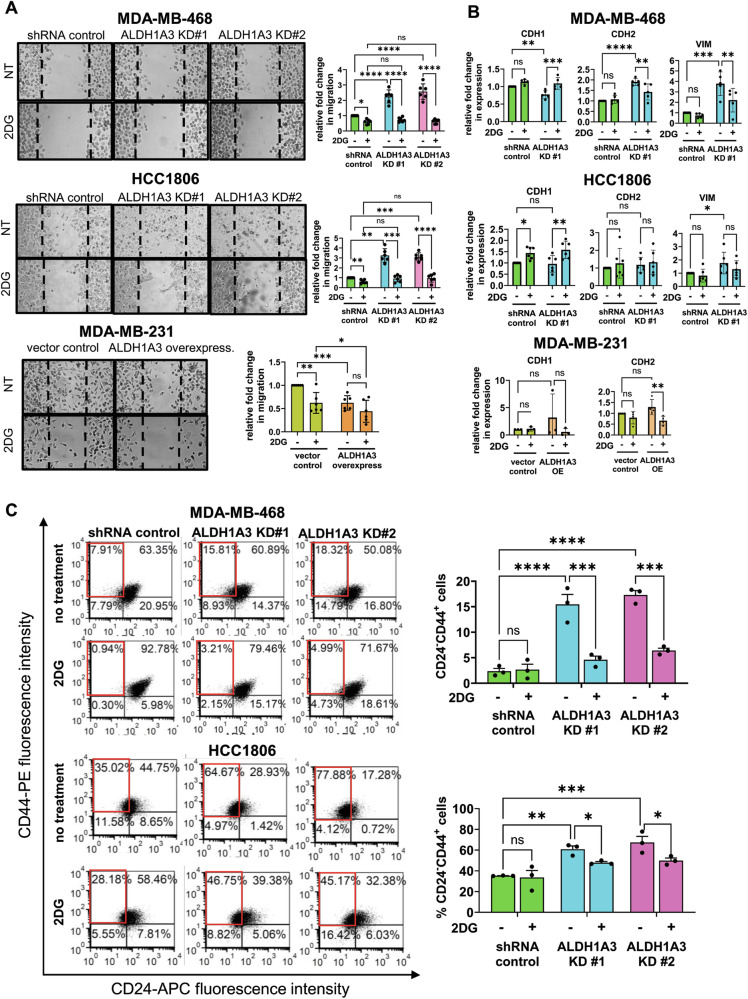
2DG inhibits the increased migration, effects on EMT/MET gene expression, and increased CD24^−^CD44^+^ population induced by reduced ALDH1A3 levels in triple-negative breast cancer cells. **A** The effect of 48 h 5 mM treatment with 2-deoxy-D-glucose (2DG) on the migration capacity of MDA-MB-468 (*n* = 5) and HCC1806 cells (*n* = 5), with or without knockdown of ALDH1A3 (by two different shRNA sequences) or in MDA-MB-231 cells (*n* = 6), with or without ALHD1A3 overexpression is determined by gap-closure assays. The images are representative of one of the biological replicates and the bar graphs show the average different biological replicates. **B** The effect of 48 h 5 mM 2DG on the relative mRNA expression CDH1, CDH2, and vimentin in MDA-MB-468 (*n* = 5) and HCC1806 cells (*n* = 6), with or without knockdown of ALDH1A3 (by two different shRNA sequences) or in MDA-MB-231 cells (*n* = 3 for CDH1, *n* = 4 for CDH2) is determined by real-time quantitative polymerase chain reaction (RT-qPCR), relative to two reference genes and the control in MDA-MB-468, HCC1806, and MDA-MB-231 cells. **C** The effect of 48 h 5 mM 2DG treatment of MDA-MB-468 cells and HCC1806 cells, with or without ALDH1A3 knockdown (*n* = 3), on the percentage of CD24^−^CD44^+^ cells is determined by flow cytometry analysis of cell stained with anti-CD24-APC conjugated and anti-CD44-PE conjugated antibody. **A**–**C** The error bars equal standard deviation and significance determined by two-way ANOVA, followed by multiple comparison post-tests (*p*-value < 0.05 = *, <0.01 = **, <0.001 = ***, <0.0001 = ****, ns = not significant).

Having observed that 2DG inhibits the increased ROS induced by low ALDH1A3, we wondered if it would also inhibit the other changes induced by low ALDH1A3 (e.g., migration, EMT/MET marker expression, CD24^−^CD44^+^ cells). We performed the gap-closure assays again, with the inclusion of 2DG treatment. This revealed that 2DG eliminates the increased migration imparted by low ALDH1A3 levels in the TNBC cells (Fig. 6A).

We next investigated if 2DG affects the expression of EMT/MET genes altered by ALDH1A3 in the breast cancer cells. In MDA-MB-468 cells, 2DG restored the reduced CDH1 levels caused by ALDH1A3 knockdown and increased the MET maker in HCC1806 cells (Fig. 6B). The EMT markers CDH2 and vimentin increased by ALDH1A3 knockdown in MDA-MB-468 were decreased by 2DG (Fig. 6B). In MDA-MB-231 cells, the increased CDH2 induced by ALDH1A3 overexpression was decreased by 2DG (Fig. 6B). Overall, these results are consistent with 2DG inhibiting the effects on EMT induced by ALDH1A3 in breast cancer.

Finally, we investigated the effects of 2DG on the percentage of CD24^−^CD44^+^ in breast cancer cells with altered ALDH1A3 levels. Treating the cells with 2DG inhibits the increase in CD24^−^CD44^+^ cells induced by ALDH1A3 knockdown in MDA-MB-468 and HCC1806 cells (Fig. 6C). Consistent with the decrease in the CSC population, 2DG treatment reduces mammosphere formation capacity of the breast cancer cells (Supplemental Fig. S4).

### Effects of ALDH1A3 and 2DG on tumor growth are reflected in the changes they induce on the CD24^−^CD44^+^ cell population and tumor-initiating cell frequency

Having characterized the in vitro effects of ALDH1A3 and 2DG on CSC populations we next wondered how this would translate into an in vivo setting. We were especially curious in the case of the MDA-MB-468 cells, which is an atypical cell line with respect to the effects of ALDH1A3 on tumor growth; to our knowledge, it is the only reported cancer cell line in which ALDH1A3 knockdown promotes tumor growth [29]. The seahorse assay (Fig. 4C) revealed low levels of aerobic glycolysis in MDA-MB-468 cells that were increased by ALDH1A3 knockdown. It suggests that increased tumor growth upon ALDH1A3 knockdown in the MDA-MB-468 cells [29], could be connected to the increased glycolysis, which is needed for in vivo tumor growth [57–61]. Conducting the tumor growth assays in the presence of glycolysis inhibitor 2DG would test this theory.

**Fig. 7:**
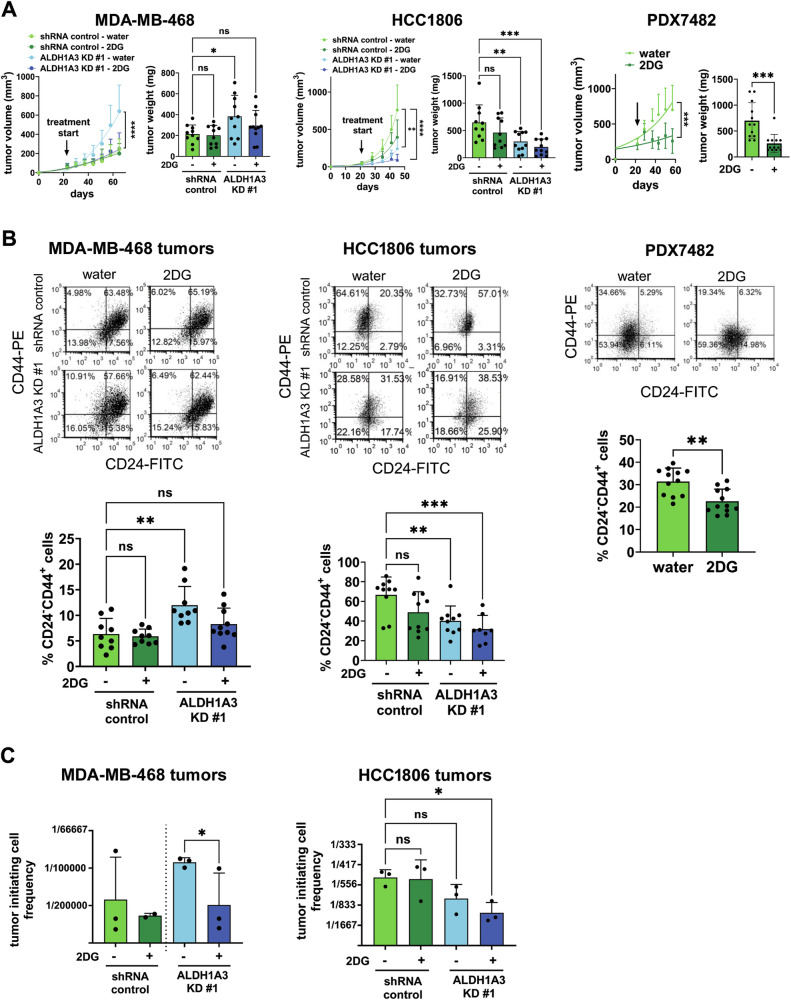
2DG and ALDH1A3 knockdown in triple-negative breast tumor xenografts alters tumor growth, the percentage of CD24^−^CD44^+^ cells, and the frequency of tumor-initiating cells. **A** MDA-MB-468 (*n* = 10-11) and HCC1806 (*n* = 10-11) tumor growth in NOD/SCID female mice, with or without ALDH1A3 knockdown, or PDX7482 (*n* = 12) and treatment with water or 0.4% w/v 2-deoxy-D-glucose (2DG) started when tumors are palpable (indicated with the arrow) in the tumor volume plot, which show weekly caliper measurements. (length X width X width /2). The data points (day versus tumor volume measurements) are graphed with a non-linear regression curve of best fit. The bar graphs show the final tumor weights from harvested tumors at experiment termination. **B** The harvested tumors from (**A**), are analyzed for percentages of CD24^−^CD44^+^ cells by flow cytometry analysis of single-cell suspensions post lysing or red blood cells and staining with anti-CD44-PE and anti-CD24-APC antibody. Staining with Fluor-488 conjugated anti-H2Kd antibody was used to eliminate mouse cells from the analysis. **C** The harvested tumors from (**A**) were analyzed for tumor-initiating cell frequency by performing a limiting dilution assay were increasing numbers of live cells isolated from three (or two for MDA-MB-468 control tumors treated with 2DG) harvested tumors were injected into the mammary fat pads of 4–6 mice and scored for tumor development (as detailed in Supplemental Tables S2 and S3). **A**–**C** The error bars equal standard deviation and significance determined by one-way ANOVA followed by multiple comparison post-tests (except the MDA-MB-468 experiment in **C**, where we just performed a *t*-test between the ALDH1A3 KD water versus 2DG treatment groups). The *p*-values are indicated as follows: <0.05 = *, <0.01 = **, <0.001 = ***, <0.0001 = ****, ns = not significant.

In agreement with our previous work, ALDH1A3 knockdown in MDA-MB-468 cells increases tumor growth (as per tumor volumes and final tumor weights), and as hypothesized, treatment of the mice with 2DG in their water blocks this effect (Fig. 7A, left). In HCC1806 cells, we found tumor growth effects in line with other studies of ALDH1A3 in cancer, where ALDH1A3 knockdown reduces tumor growth (as per tumor volumes and tumor weights, Fig. 7A, center). The inclusion of 2DG treatment made this tumor reduction more significant (Fig. 7A, center). We also extended the 2DG treatment analysis to include a TBNC PDX and found that the PDX7482 tumor volumes and final tumor weights were reduced by the 2DG treatment (Fig. 7A, right).

We analyzed the harvested tumors at the termination of the experiment to evaluate the effect of ALDH1A3 knockdown and 2DG treatment on the percentage of CD24^−^CD44^+^ cells and the tumor-initiating cell frequency. Flow cytometry analysis of the processed tumors revealed that in the MDA-MB-468 tumors, ALDH1A3 knockdown increased the percentage of CD24^−^CD44^+^ cells and 2DG blocked this effect (Fig. 7B, left). These results mirrored the tumor growth effects we observed in the MDA-MB-468 tumors in Fig. 7A. For the HCC1806 tumors, we again observed results that mirrored the tumor growth effects in Fig. 7A, where ALDH1A3 knockdown decreased the percentage of CD24^−^CD44^+^ cells in the HCC1806 tumors (Fig. 7B, center), and 2DG made this tumor reduction more significant (Fig. 7B, center). Surprisingly, the in vivo results for HCC1806 tumors differ from the in vitro results we observed in Fig. 1 and suggest that ALDH1A3 can conversely promote the abundance of the CD24^−^CD44^+^ population under the selective pressures encountered in in vivo tumor microenvironment. Finally, for TNBC PDX7482, the percentage of CD24^−^CD44^+^ cells was reduced by 2DG treatment (Fig. 7B, right) and again reflected the tumor growth reduction we observed (Fig. 7A, right).

We were able to harvest sufficient cells from a few of the MDA-MB-468 and HCC1806 tumors to complete a limiting dilution assay where serial dilutions of the cells were injected into the mammary fat pads of new NOD/SCID female mice. The mice were scored for tumor development and based on the number of mice that developed tumors for each dilution, the tumor-initiating cell frequency was calculated (Fig. 7C, Supplemental Tables S2 and S3). This showed that for the MDA-MB-468 tumors, the ALDH1A3 knockdown tumors had the greatest number of tumor-initiating cells, and 2DG treatment blocked this effect. Unfortunately, we could only calculate the tumor-initiating cell frequency for two tumors in one group; the shRNA control tumors treated with the 2DG group. This limited our ability to perform statistical analyses on all four groups, so we only performed a *t*-test on the ALDH1A3 knockdown no-treatment tumors versus the 2DG treatment groups. The tumor-initiating cell frequency we observe in MDA-MB-468 tumors (Fig. 7C, left) reflects CD24^−^CD44^+^ cell percentages (Fig. 7B, left) and tumor growth (Fig. 7A, left) data, and provides a probable explanation for why MDA-MB-468 cells, ALDH1A3 knockdown results in increased tumor growth [29]. For the HCC1806 tumors, the ALDH1A3 knockdown tumors treated with 2DG had the fewest tumor-initiating cells (Fig. 7C, right). The tumor-initiating cell frequency for the HCC1806 tumors (Fig. 7C, right) reflects the tumor growth (Fig. 7A, center) and CD24^−^CD44^+^ cell percentages (Fig. 7B, center) we observed. Taken together, the in vivo data suggests that ALDH1A3’s effects on tumor growth are reflected in the changes it induces in the CD24^−^CD44^+^ cell population and the overall tumor-initiating cell frequency, which are also susceptible to the glycolysis inhibitor 2DG.

## Discussion

The results presented in this study provide a comprehensive analysis of the role of ALDH1A3 in TNBC, unraveling its influence on CSC populations, partial EMT, and glucose metabolism. Breast cancer metastasis and recurrence are linked to high abundances of the two distinct breast CSC populations defined by CD24^−^CD44^+^ cell surface markers and high ALDH activity [7, 8, 35, 37, 38, 75]. The ability of breast CSCs to transition between these two CSC populations depending on stressors that favor the phenotype of one CSC population over the other, allows the tumor to survive and thrive under the harsh conditions of the tumor microenvironment [2]. Therefore, determining the factors that regulate the shift between these two CSC populations is critical if tumors are to be eradicated and recurrence eliminated. A key finding in this study is the modulation of the two distinct CSC populations by ALDH1A3. High ALDH1A3 increases the ALDH^+^ population, consistent with previous studies linking ALDH1A3 to the breast CSC population [12, 13, 16, 21, 26]. However, the unexpected relationship of ALDH1A3 with the CD24^−^CD44^+^ cell population provides new information about the balance that exists between these two distinct tumor-promoting CSC populations. It also provides an explanation for the tumor growth-inhibiting effects induced by ALDH1A3 in MDA-MB-468 cells (especially with the observed shift in glucose metabolism) that we could not explain before [29].

RT-qPCR revealed that ALDH1A3 exerts its effects at the transcriptional level influencing the expression of CD24, CD44, EMT, and glucose metabolism genes. ALDH1A3’s suppression of CD44 expression and induction of CD24 expression suggest a regulatory role in the phenotypic characteristics of TNBC cells. Moreover, we showed the connection with retinoic acid signaling in mediating these ALDH1A3 effects. Treating the cells with the ALDH1A3 substrate retinal revealed that high ALDH1A3 levels are necessary for retinal to influence the CD24^−^CD44^+^ cell population. This aligns with prior knowledge of ALDH1A3’s involvement in retinoic acid signaling [29, 39] and adds a layer of complexity to its regulatory functions in TNBC.

Increasing evidence is demonstrating the critical importance of the partial or hybrid EMT phenotype in metastasis. For example, tracing experiments elegantly showed that only primary mammary tumor cells that had undergone partial EMT contribute to lung metastasis and chemoresistance, while the cancer cells that have undergone full EMT and have a mesenchymal phenotype fail to colonize the lungs [76]. Here we found that ALDH1A3 induces a mixed EMT/MET phenotype in breast cancer cells that have increased invasion but decreased migration. Molecular analyses revealed alterations in the levels of focal adhesion proteins, FAK and Src, and the proteins of the adherens junctions, E-cadherin and N-cadherin. The observed gene expression changes, with concurrent alterations in EMT and MET markers, further emphasize the role of ALDH1A3 in driving a phenotypic shift in TNBC cells that was an intermediate/partial EMT-MET phenotype. Single-cell RNA sequencing revealed that breast cancer cells express both epithelial and mesenchymal markers suggesting an intermediate hybrid EMT phenotype [77]. Deshmukh et al., further report that the hybrid EMT state is associated with poor patient prognosis [77]. Partial or hybrid/intermediate EMT is viewed as a plastic transient state, mediated by cancer cells that express both epithelial and mesenchymal markers and these cancer cells have increased capacity for tumorigenicity, invasion and metastasis, stemness, and therapy resistance [78]. The mixed decreased migration/increased invasion ALDH1A3-induced phenotypic changes, and the impact of ALDH1A3 on both EMT and MET markers, suggest that ALDH1A3 is a key player in determining the plastic hybrid EMT-MET state in breast cancer. It will be important to investigate these effects of ALDH1A3 EMT-MET in other cancers. At least in colon cancer, ALDH1A3 increases invasion by upregulating EMT-inducing transcription factor zinc finger E-box binding homeobox 1 (ZEB1) and SNAI2 and by inhibiting MET-promoting miR-200 family microRNAs [79].

EMT and glucose metabolism are well-established to be linked in cancer, with crosstalk between the two pathways [80]. Metabolic reprogramming and glycolysis are often necessary for EMT [81]. Analysis of more than 180 cancer cell datasets and showed that EMT is generally positively correlated with glycolysis and negatively correlated with oxidative phosphorylation across cancer, with detectable patterns of metabolic plasticity as cancer cells transition through hybrid EMT states [82]. Here, we add to this body of work by investigating the effects of ALDH1A3 on the CD24^−^CD44^+^ population and uncover ALDH1A3’s influence on EMT/MET, concomitant with its promotion of oxidative phosphorylation over glycolysis.

ALDH1A3 decreases aerobic glycolysis and lactate production while increasing ATP synthase activity of oxidative phosphorylation in breast cancer cells. In MDA-MB-468 cells, this translated to changes in ATP production from glycolysis versus oxidative phosphorylation. Treatment with the glycolysis inhibitor 2DG reverses the increased ROS resulting from reduced ALDH1A3, indicating a potential link between ALDH1A3, ROS, and the balance of glucose metabolism pathways. The relationship between ROS and glycolysis is well-established. ROS induces glycolysis by multiple mechanisms, including induction of glycolysis pathway activator transcription factor HIF1α and upregulation of glycolytic enzymes, including hexokinase type 2 (HK2), lactate dehydrogenase (LDH) and glucose transporter 1 (GLUT1) [83]. ROS can also upregulate various kinases, including energy-sensing AMP-protein kinase (AMPK), phosphatidylinositol 3-kinase (PI3K), and protein kinase B (PKB, also known as AKT), which induce expression, and activation, of key enzymes in the glycolysis pathway [84–87]. It is noteworthy that analyses of the role of ALDH1A3 in glucose metabolism in pancreatic cancer showed differing results, where ALDH1A3 promotes pancreatic cancer progression and metastasis by increasing aerobic glycolysis [88]. In pancreatic cancer cells, ALDH1A3 increases the expression of HK2 by its activation of the PI3K/AKT/mTOR pathway [88]. Therefore, there are cancer-specific effects in terms of the role of ALDH1A3 in glycolysis and oxidative phosphorylation and we cannot generalize the effects we observe in breast cancer to other cancer types.

The integration of 2DG here revealed its inhibitory effects on ALDH1A3-induced phenotypes. 2DG not only reverses the increased migration observed with low ALDH1A3 but also impacts the expression of EMT/MET markers and the percentage of CD24^−^CD44^+^ cells. The effects of 2DG on the cell lines cannot be fully explained by its inhibition of glycolysis. For example, 2DG also causes a condensed chromatin state and histone deacetylation in cancer cells [89], which could lead to altered gene expression. Hence, the effects of 2DG on the EMT/MET phenotypes of cells could be related to epigenetic mechanisms.

A key observation was the heterogeneity in responses between cell lines in the in vitro versus in vivo setting. The in vivo results underlie the selective pressures of the tumor microenvironment that cannot be necessarily predicted by in vitro findings. Previous analyses showed that mesenchymal breast CSC (i.e., CD24^−^CD44^+^ cells) rely on aerobic glycolysis and inversely epithelial breast CSCs (i.e., ALDH^+^ or Aldefluor^high^) favor oxidative phosphorylation [10]. Furthermore, 2DG, H_2_O_2_, and hypoxia promote the transition toward the epithelial ALDH^+^ population and ROS inhibition reverses this effect by activation of the AMPK-HIF1α axis [10]. Others have similarly shown that 2DG inhibits the CD24^−^CD44^+^ population [90]. CD44 could also be targeted directly and there has been much interest in developing anti-CSC therapies by targeting CD44 [91], but it will be important to determine how targeting CD44 would affect the ALDH^+^ CSC population. Here, we add a new key element to our understanding of the plastic metabolic switch between the two CSC populations by showing that ALDH1A3 promotes the cancer-promoting phenotypes associated with the epithelial ALDH^+^ CSC phenotype, while inversely inhibiting the CD24^−^CD44^+^ cells associated with the mesenchymal breast CSC populations.

Molecular profiling has revealed the heterogeneity of TNBC and its multiple subtypes [92]. Most TNBCs fall within the basal-like subtypes, which also have high ALDH1A3 [29, 93]; HCC1806 and MDA-MB-468 cells fit within the basal-like categories [92]. In contrast, the mesenchymal or claudin-low TNBC subtypes have higher percentages of CD24^−^CD44^+^, and MDA-MB-231 cells are claudin-low/mesenchymal and have high percentages CD24^−^CD44^+^ cells [47, 92, 94, 95]. Hence, an expansion of this analysis to include additional cell lines representing the different TNBC molecular subtypes would clarify any subtype-specific effects that are specific to the role of ALDH1A3 in determining the balance of the two distinct CSC populations and the partial EMT phenotype. Although not the focus of this study, we did include some analyses on ALDH1A1 that suggest it could also affect the balance of ALDH^+^ versus CD24^−^CD44^+^ breast cancer cells. Undoubtedly multiple factors can affect the maintenance of these distinct two CSC populations beyond ALDH1A3 (e.g., NRF2, ITGB4 [10, 45, 71]), and ALDH1A1 is another likely candidate. Along with the increased expression of EMT genes, ALDH1A1 is expressed in the hybrid ALDH^+^CD24^−^CD44^+^ cells [43, 47]. It would be valuable to increase our understanding of the role of both ALDH1A3 and ALDH1A1 in the regulation of the hybrid EMT phenotype in ALDH^+^CD24^−^CD44^+^ cells.

## Conclusions

This study provides a comprehensive exploration of ALDH1A3’s multifaceted impact on breast cancer, elucidating its roles in CSC maintenance and tumor heterogeneity, EMT and MET, and metabolic reprogramming. Therefore, ALDH1A3 emerges as a key player in a few “hallmarks of cancer” [96, 97]. The integration of in vitro and in vivo analyses enhances the translational relevance of the findings. The study’s insights into the interconnected pathways influenced by ALDH1A3 contribute to our evolving understanding of TNBC biology and open avenues for further research aimed at refining targeted therapeutic strategies. The study’s integration of 2DG as a potential therapeutic intervention aligns with emerging strategies targeting metabolic vulnerabilities and CSCs in cancer [98].

## Materials and methods

### Cell lines, patient-derived xenograft, cell culture, and reagents

HCC1806, MDA-MB-231, and MDA-MB-468 cell lines were purchased from the American Type Culture Collection (ATCC, Manassas, VA). HCC1806 cells were grown in RPMI-1640 supplemented with 10% fetal bovine serum (FBS), and antibiotic-antimycotic (AA). MDA-MB-231 and MDA-MB-468 cell cultures were maintained in DMEM media supplemented with 10% FBS and AA (media components are all from ThermoFisher Scientific, Invitrogen, Carlsbad, CA, USA). ALDH1A3 knockdown and overexpression clones were generated as described before [29]. The clones were maintained in the respective media containing 0.25 µg/mL puromycin (Millipore Sigma, Oakville, Canada). The cell lines have been authenticated in the past three years by isolation of genomic DNA and performance of short tandem repeat profiling technology by Applied Biological Materials Inc. (Richmond, Canada). We confirm that the cells are free of mycoplasma contamination by regularly performing MycoAlert® Mycoplasma Detection Kit (Lonza, Kingston, Canada).

The cryopreserved early passaged tumor pieces of TNBC patient-derived xenograft (PDX) 7482 (provided by Michael Lewis, Baylor College of Medicine, Houston, TX, USA) originated from a grade 3, stage 2 primary tumor, breast carcinoma (https://pdxportal.research.bcm.edu) [99]. As described by Zhang et al. [99], PDXs were generated from tumor samples of consenting patients recruited from clinics in the Baylor College of Medicine (BCM) Breast Center (Houston, TX) and Ben Taub General Hospital (Houston, TX) under Institutional Review Board-approved protocols. For work with the PDX, approval was obtained from the Dalhousie University Committee on Laboratory Animals, which adheres to the ethical standards according to the Declaration of Helsinki and to the Canadian Council on Animal Care standards (protocol numbers 19-013 and 21-011). Prior to experimentation, the cryopreserved tumor pieces were revived and surgically implanted in the mammary fat pad of a non-obese diabetic severe combined immunodeficient (NOD-SCID) female mouse (Charles River Laboratories, Senneville, Canada) for expansion approximately 5 weeks. The resulting expanded tumor tissue was used for subsequent experiments. For isolation of cells for experiments, the harvested tumors were minced and digested in 225 U/mL collagenase type III (BioShop Canada Inc., Burlington, Canada) in a 37 °C incubator for 1 h. The cells were strained through a 70 μm cell strainer and washed with cold phosphate-buffered saline (PBS). Single-cell suspensions of tumors were made, and red blood cells were lysed by incubating in red blood cell lysis buffer (157 mM NH_4_Cl, 20 mM KHCO_3_, pH 7.4, Millipore Sigma).

### Flow cytometry

Cell analysis of distinct stained cell populations in cultured cells and xenografted harvested tumors was completed using a FACSCelesta (BD BioSciences, Mississauga, ON, Canada) followed by analysis using FCSExpress 4 RE analysis software (De Novo Software, Pasadena, CA, USA).

The abundance of CD24^−^CD44^+^ cells in cultures and xenograft tumors was determined by staining PBS-washed 2 × 10^5^ cells in FACS buffer (1 mM EDTA solution containing 1% FBS) containing allophycocyanin (APC) conjugated anti-CD24 (cat# 138506, BioLegend, San Diego, CA) and phycoerythrin (PE) conjugated anti-CD44 (cat# 12-0441-82, eBiosciences, ThermoFisher Scientific). Cells were also stained with isotype APC mouse IgG2a kappa isotype control (cat# 400219, Biolegend) or Rat IgG2b kappa isotype control (eB149/10H5), PE (cat# 12-4031-82, eBioscience) for setting flow cytometry gates. Additionally, for sorting the dissociated cells of xenografted tumors, the cells were also stained with Alexa Fluor-488 conjugated anti-H2Kd antibody (cat# 116510, Biolegend) or its negative isotype control mouse (SJL) IgG2a kappa (cat# 400233, Biolegend) to remove mouse cells. The cells were incubated in the presence of antibodies for 30 min and occasionally mixed and resuspended with cell death marker 7AAD to exclude dead cells before analysis. To evaluate the effects of retinal or glycolysis inhibition, the cells were pretreated with 100 nM retinal (Millipore Sigma) or 5 mM 2-deoxy-D-glucose (2DG, Millipore Sigma) for 24 h or 48 h, respectively.

To evaluate reactive oxygen species levels (ROS) in the cells, 2′,7′-dichlorodihydrofluorescein diacetate (H_2_DCFDA) (ThermoFisher Scientific) assays were performed using flow cytometry. ALDH1A3 knockdown cultures (MDA-MB-468 and HCC1806) and ALDH1A3 overexpression cultures (MDA-MB-231) along with their respective control (2 × 10^5^ cells from each clone) were seeded in 6-well plates. Adherent cells were incubated in the presence or absence of 5 mM 2DG for 48 h. Cells were then thoroughly washed with warm PBS and incubated with 5 μM DCFDA (ThermoFisher Scientific, Mississauga, ON) in phenol red-free, serum-free medium for 45 min. At the end of the incubation, the cells were washed with warm PBS again to remove excess stain and the DCFDA fluorescence intensity of the cells was measured by flow cytometry.

### Quantitative polymerase chain reaction

Cells were harvested from 6-well plates using 1 mL TRIzol reagent per well (Life Technologies Inc. Burlington, ON). In experiments where cells were treated with 5 mM 2DG, the cells were cultured for 48 h prior to harvesting. RNA was extracted using a PureLink RNA Mini Kit (Life Technologies Inc., Burlington, ON) according to the manufacturer’s instructions and RNA was quantified using a SpectraMax m2 Microplate Reader (Molecular Devices, San Jose, CA) equipped with SoftMax Pro software (Molecular Devices, San Jose, CA). According to the manufacturer’s instructions, complementary DNA (cDNA) was made by reverse transcription of RNA using iScript RT Supermix (Bio-Rad, Mississauga, ON) in a T100 Thermal Cycler (Bio-Rad, Mississauga, ON). cDNA was amplified with SYBR Green Supermix (Bio-Rad, Mississauga, ON) using a CFX96 or CFX384 RT-qPCR thermocycler (Bio-Rad, Mississauga, ON), and fold expression of genes of interest were determined using the ΔΔq value with at least two reference genes (primers listed in Supplemental Table S1).

### EMT score

The EMT scores of the breast cancer patient tumor samples from the Cancer Genome Atlas Breast Invasive Carcinoma (TCGA BRCA, Cell 2015) and METABRIC datasets were calculated using gene expression data accessed via cBioportal [100, 101] and applying the EMT score metric developed by Tan et al. [56]. Briefly, the score was calculated by evaluating cumulative distribution functions (CDFs) for epithelial and mesenchymal gene signatures within a given sample and consists of values for over 300 genes. The distance between these signatures was determined by measuring the maximum separation between their respective CDFs. This value serves as the test statistic for the subsequent two-sample test to calculate the EMT score. The resulting score falls within the range of −1 to +1, where a positive EMT score indicates a mesenchymal phenotype and a negative EMT score signifies an epithelial phenotype in the sample.

### Western blot analysis

Cells were harvested and incubated in radioimmunoprecipitation (RIPA) buffer (50 mM Tris at pH 7.5, 0.25% sodium deoxycholate (w/v), 1% Nonidet P-40 (v/v), 150 mM NaCl, 1 mM EDTA) containing freshly added protease and phosphatase inhibitor cocktail (Millipore Sigma) on ice for 2 min. Cells were disrupted with a sonicator and then centrifuged at 10,000 × *g* for 10 min and debris removed. Total protein concentration was determined using a BCA Colorimetric protein quantification assay. Equal amounts of protein in Lamelli buffer with β-mercaptoethanol were loaded onto 10% TGC Stain-free FastCast Acrylamide gels (Bio-Rad, Mississauga, Canada) and electrophoresed. Proteins were transferred onto nitrocellulose membranes (Trans-Blot Turbo PVDF membrane) and blocked for one hour with 5% skim milk. Blots were probed overnight with either total steroid receptor coactivator (Src, cat# 2108S), focal adhesion kinase (FAK, cat# 3285S) antibody, phospho Src (Tyr 416, cat# 6943S), or phospho FAK (Tyr 397, cat# 3283S, Cell Signaling Technology, New England Biolabs Ltd., Whitby Canada). Blots were washed with TBST (tris-buffer saline with 0.1% Tween20) and probed with the according to HRP-conjugated secondary antibody (Jackson Immunoresearch Labs, West Park, PA, USA) for one hour, then washed with TBST. ECL substrate (1:1 peroxide solution: luminol solution, Bio-Rad) was added to the blots, which were then imaged using a ChemidocTouch™ imaging system (Bio-Rad). Densitometric analysis on at least three biological replicates was performed using Image lab (Bio-Rad) and relative quantity was analyzed against the total protein. One-way ANOVA was performed for statistical analysis. The figure panel was designed using QuickFigures plugin of ImageJ [102].

### Immunofluorescence analysis

Cells were grown on glass slides up to 60–80% confluency and fixed with 1% paraformaldehyde in PBS for 1 h and blocked in 3% bovine serum albumin (BSA) in PBS for 1 h. Cells were stained overnight with polyclonal rabbit anti-E-Cadherin antibody (cat# PA5-80457, ThermoFisher Scientific, Invitrogen) and monoclonal mouse anti-N-cadherin (cat# 33-3900, ThermoFisher Scientific, Invitrogen) at 1:100 in 0.3% BSA in PBS. Slides were also stained with negative isotype controls rabbit IgG cat# PI31235 or mouse IgG1 kappa antibodies (cat# 501129514, ThermoFisher Scientific, Invitrogen). Washed slides were stained with secondary Alexa Fluor-488 conjugated anti-rabbit (cat# 111-545-003, Jackson Immunoresearch Labs) and CyTM3 conjugated anti-mouse (cat# 115-165-003, Jackson Immunoresearch Labs) antibodies were used at 1:100 in 0.3% BSA in PBS for 1 h. Nucleus was counterstained with DAPI (Sigma) in 0.3% BSA in PBS 0.1% Tween. Stained cells were imaged with a Zeiss LSM 880 confocal microscope (Carl Zeiss, Jena Germany) using a 40× oil immersion objective at different locations on the slide. Images from biological replicates were analyzed using ImageJ. Regions of interest (ROIs) were established using cell boundaries and the individual red and green fluorescence were measured in individual cells. Regions without cells were used as blank.

### Gap-closure assay

The effects of ALDH1A3 knockdown, overexpression, and 2DG treatment on cell migration were determined using gap-closure assays. ALDH1A3 shRNA knockdown and scramble shRNA control HCC1806 (1 × 10^4^ cells) and MDA-MB-468 (2 × 10^4^ cells) cells as well as overexpression and empty vector of MDA-MB-231 (1 × 10^4^ cells) cells were cultured in silicon 2-well cell culture inserts. Adherent cells were treated with 10 µg/mL mitomycin C for 2 h to inhibit cell proliferation and let to recover overnight. The migration of the cells was monitored in the presence or absence of 5 mM 2DG for 48 h. Culture inserts were removed, and the gap was photographed (*t* = 0 h). The gap was periodically photographed until it was completely closed by the empty vector control cells (HCC1806 and MDA-MB-231 cells at 22 h; MDA-MB-468 cells at 36 h).

### Trans-well migration/invasion assay

The effects of ALDH1A3 knockdown, ALDH1A3 overexpression, and 2DG treatment on cell migration and invasion were investigated with trans-well invasion assays using a trans-well cell migration apparatus. HCC1806, MDA-MB-468, and MDA-MB-231 control and knockdown or overexpression cells were treated with 5 mM 2DG for 48 h and cells were harvested and counted at the end of the incubation. HCC1806 cells (10^6^ cells/mL) were resuspended in serum-free RPMI medium, MDA-MB-468 cells (10^6^ cells/mL) in DMEM medium supplemented with 1% FBS, and MDA-MB-231 cells (10^6^ cells/mL) were resuspended in serum-free DMEM medium. The migration of the HCC1806, MDA-MB-468, and MDA-MB-231 cells through an 8 μm porous membrane toward RPMI medium supplemented with 10% FBS and DMEM medium containing 20% FBS and DMEM medium supplemented with 10% FBS respectively was determined. The porous membrane was coated with 1 mg/mL gelatin to evaluate the invasiveness of the cells.

### Glycolysis extracellular acidification assay

Effects of ALDH1A3 on glycolysis were assessed with the fluorescent Glycolysis Assay [Extracellular activation] (cat# ab197244, Abcam, Toronto, ON). MDA-MB-468 (3 × 10^4^ cells), HCC1806 (3 × 10^4^ cells) with or without knockdown, and MDA-MB-231 (3 × 10^4^ cells) (with or without overexpression) were seeded in black-walled black bottom 96-well plates. Adherent cells were subjected to overnight CO_2_ purge and some wells were treated with 2DG (5 mM) for 48 h. The cells were incubated in the glycolysis assay buffer at 37 °C and fluorescence at Ex/Em = 380/615 nm was recorded at 30-s intervals for 1 h using a microplate reader (Molecular Devices, San Jose, CA). The glycolytic activity was normalized to cell number.

### ATP synthase activity assay

The effect of ALDH1A3 on the ATP synthase activity of breast cancer cells was measured using an absorbance microplate assay (cat# ab109714, Abcam, Toronto, ON). Proteins (5 mg/mL) isolated from MDA-MB-468 and HCC1806 cells, with or without ALDH1A3 knockdown, and MDA-MB-231 cells, with or without ALDH1A3 overexpression, were incubated with 1/10 volume detergent solution. The supernatant was separated by centrifuging at 16,000 × *g* for 20 min and incubated for 3 h in provided microplate wells to immobilize the enzymes. At the end of the incubation, a phospholipid mix was added and incubated for another 45 min. After adding the reagent mix, the absorbance of each well was measured at 340 nm, 1 min intervals for 30 min at 30 °C. The rate of ATP synthase activity was measured using the equation below.

$$Rate\left(\frac{mOD}{\min }\right)=\frac{(Absorbance\,1-Absorbance\,2)}{Time\,({\min })}$$

### Seahorse XF real-time ATP rate assay

The effect of ALDH1A3 knockdown in MDA-MB-468 and HCC1806 cells, or ALDH1A3 overexpression in MDA-MB-231 on real-time oxygen consumption rate (OCR) and H^+^ production (extracellular acidification rate, EACR) and the kinetic quantification of ATP production, for both oxidative phosphorylation (mitochondria) ATP and glycolytic (glyco) ATP production rates, and the total ATP production rates was determined using a Seahorse XFe96 Extracellular Flux Analyzer (Agilent Technologies Canada Inc., Toronto, Canada) with a Seahorse XF Real-Time ATP Rate Assay (Agilent Technologies Canada Inc.). As per the manufacturer’s protocol, optimized cell numbers were seeded into a Seahorse XFe96 well culture plate. We seeded 25,000 cells per well for MDA-MB-468 and HCC1806 cells and 35,000 cells per well for MDA-MB-231 cells. The cells were cultured for 24 h at 37 °C in a 5% CO_2_ humidified chamber. Following incubation, cells were washed twice, and media was replaced with Seahorse XF DMEM (or RPMI for HCC1806 cells) media supplemented with Seahorse XF glucose (10 mM), Seahorse XF pyruvate (1 mM), and Seahorse XF L-glutamine (2 mM) then incubated at 37 °C in a non-CO_2_ incubator for 1 h prior to assay. The ECAR and OCR flux were measured for three time points under basal conditions and another three time points after the sequential addition of mitochondrial inhibitors oligomycin and rotenone/antimycin A. The total cellular ATP Production Rates and mitoATP and glycoATP production rates were determined as per the manufacturer’s protocol. To compare among groups, data are presented as OCR or ECAR in pmol/min or mpH/min respectively.

### Mouse experiments

All mouse experiments were conducted in accordance with the ethical standards according to the Declaration of Helsinki and to the Canadian Council on Animal Care standards and protocols (19-013 and 21-011) approved by the Dalhousie University Committee on Laboratory Animals. Six to 12-week-old non-obese diabetic severe combined immunodeficient (NOD-SCID) female mice from Charles River Laboratories (Senneville, QC) were used for all the in vivo experiments in this study. The mice were orthotopically implanted in the 5th mammary fat pad with either 2 × 10^6^ MDA-MB-468 (control or knockdown), 1 × 10^3^ HCC1806 (control or knockdown) cells or with 2 × 10^5^ cells of PDX7482. The cells were admixed in a 1:1 ratio with phenol red-free high concentration Matrigel (Corning, ThermoFisher). Resulting tumor growth was quantified (mm^3^; length × width × width/2).

Once tumors were palpable, the mice were divided into two groups by randomly allocating to the treatment groups, and the one investigator measuring and harvesting tumors was blinded to the group allocations for the entire duration of the experiment. For the treatment group, the regular drinking water was replaced with 0.4% w/v 2DG (Millipore Sigma), dissolved in water. At termination, the tumors were harvested and weighed. Tumors were minced and single-cell suspensions generated as described above. Cells were counted and 2 × 10^5^ cells were stained for flow cytometry analysis as described in the below section.

Alternatively, a limiting dilution assay was performed by injecting increasing numbers of live cells into the mammary fat pads of female mice. For harvested MDA-MB-468 tumors, we injected 5000, 50,000, and 500,000 cells into up to 4 mice per dilution for two to three different tumors per treatment group, in the 5th mammary fat pad per mouse. For harvested HCC1806 tumors, we injected 10, 100, 1000 cells into up to 6 mice per dilution from three different tumors per treatment group, in the 5th mammary fat pad per mouse. The mice were monitored daily for up to two months and scored for tumor development by an investigator who was blinded to the groups (Supplemental Tables S2 and S3, animal numbers are included). At termination, the mice that developed and did not develop tumors for each dilution were noted and the tumor-initiating cell frequency was determined using the Extreme Limiting Dilution Analysis tool, by inputting the cell number injected, total number of mice, and the number of mice that developed tumors for each concentration of cell number that was injected into the mice (https://bioinf.wehi.edu.au/software/elda/) [103].

### Statistical analysis

All statistical analyses were performed using GraphPad Prism. The exact sample size (*n*) for each experimental group is provided in the figure legends and “Results” section. Statistical significance was assessed using Student’s *t*-test, Mann–Whitney U test, or one-way or two-way ANOVA depending on the data distribution and experimental design, as stated in each figure legend. Tests were two-sided unless otherwise stated and the estimation of variation was determined. *P*-values are reported to indicate the significance of the results, with *p* < 0.05 considered statistically significant unless otherwise specified.

## Supplementary information

Supplemental Figures and Tables

## Data Availability

Supplemental Figures and Tables are available in the Supplemental Figures and Tables file and TCGA and METABRIC patient tumor data can be accessed at the cBioportal site.
