# Supplementary material for: ALDH1A3 is the switch that determines the balance of ALDH+ and CD24−CD44+ cancer stem cells, EMT-MET, and glucose metabolism in breast cancer
Source: Oncogene. 2024 Sep 9;43(43):3151–69. doi: 10.1038/s41388-024-03156-4 (PMC11493680; doi:10.1038/s41388-024-03156-4)
Supplement: Supplementary file 1 — Supplemental Figures and Tables [file 41388_2024_3156_MOESM1_ESM.pdf]

SUPPLEMENTAL MATERIALS and METHODS

Western blot analysis of ALDH1A3 or ALDH1A1

Western blots were performed as described in the Materials and Methods section of the manuscript, but using primary mouse monoclonal antibody against ALDH1A3, clone OTI4E8 (catalog number TA502841, Origene, Rockville, MD, USA). ALDH1A1 protein was detected with BD Transduction^{TM} laboratories purified mouse anti-ALDH (Clone 44/ALDH (RUO), (catalog number 611194, BD Biosciences Mississauga, ON, Canada).

ALDH1A1 overexpression

ALDH1A1 overexpression in MDA-MB-231 cells was achieved as previously described.[1,2]

Flow cytometry

Flow cytometry analyses were performed as described in the Materials and Methods section of the manuscript, with stained cell analyzed using a FACSCelesta (BD BioSciences, Mississauga, ON, Canada) system. Additionally, ALDH+ cells were quantified using the Aldefluor assay (STEMCELL Technologies, Vancouver, Canada) as per the manufacturers' instructions.

To assess the effects of retinoic acid, cells were pre-treated with 100nM retinoic acid (Millipore Sigma) for 24h and then analyzed for CD24 and CD44 cell surface levels on cells as per the Material and Methods section of the manuscript.

Addition, to confirm that the increased fluorescence associated with ALDH1A3 knockdown as detected by the 2',7'-dichlorodihydrofluorescein diacetate (H_2DCFDA) assay is due to increased reactive oxygen species (ROS), we seeded HCC1806 (2×10^5 cells from each clone)

in 6-well plates. Adherent cells were incubated in the presence or absence of 5mM NAC (Millipore Sigma) for 24h and analyzed as described in the Materials and Methods section of the manuscript.

Cell proliferation by CellTrace Violet staining

HCC1806 breast cancer cells were seeded in 6-well plates at 50,000 cells/well density. Cells were incubated overnight to allow cell adhesion. Cells were then stained with 1uM CellTrace Violet^{TM} (ThermoFisher Scientific, Invitrogen) for 20 minutes as per manufacturer's protocol. For the non-proliferation control, well were harvested and fixed in 1% paraformaldehyde without continued culturing. The remaining stained cells were incubated for 72 hours to allow for cell proliferation and at the end of the incubation period, cells were stained with allophycocyanin (APC) conjugated anti-CD24 (BioLegend) and phycoerythrin (PE) conjugated anti-CD44 (eBioscience, ThermoFisher), or isotype negative controls (APC mouse IgG2a kappa isotype control, Biolegend, or Rat IgG2b kappa isotype control (eB149/10H5), PE, eBioscience, ThermoFisher). The cells were incubated in the presence of above-described antibodies for at 30 min and occasionally mixed. Dead cells were excluded from the analysis by staining with 7-Aminoactinomycin D (7AAD) dead cell marker. Cells were analyzed using a FACSCelesta system. $CD24^{-}CD44^{+}$ and non-$CD24^{-}CD44^{+}$ cells were gated using isotype controls and their proliferation capacity (divivisons) was measured by quantifying the percentage of cells in each peak. The data were analyzed using FCSExpress 7 analysis software (De Novo Software).

Mammosphere formation assay

The effect of ALDH1A3 knockdown, or 2DG treatment on mammosphere formation was assessed in HCC1806 cells. The cells were treated with 5mM 2DG for 48 h and the 1000 cells were washed

and were re-cultured in mammosphere media (STEMCELL Technologies) in 24 well ultra-low adherent plates (ThermoFisher Scientific), and 5 days later the resulting cultures were imaged and spheroids larger than 100µm counted.

REFERENCES

1 Marcato P, Dean CA, Liu R-Z, Coyle KM, Bydoun M, Wallace M *et al.* Aldehyde dehydrogenase 1A3 influences breast cancer progression via differential retinoic acid signaling. *Mol Oncol* 2015; **9**: 17–31.

2 Thomas ML, de Antueno R, Coyle KM, Sultan M, Cruickshank BM, Giacomantonio MA *et al.* Citral reduces breast tumor growth by inhibiting the cancer stem cell marker ALDH1A3. *Mol Oncol* 2016. doi:10.1016/j.molonc.2016.08.004.

3 Zou Z, Ohta T, Oki S. ChIP-Atlas 3.0: a data-mining suite to explore chromosome architecture together with large-scale regulome data. *Nucleic Acids Res* 2024; **52**: W45–W53.

SUPPLEMENTAL FIGURES

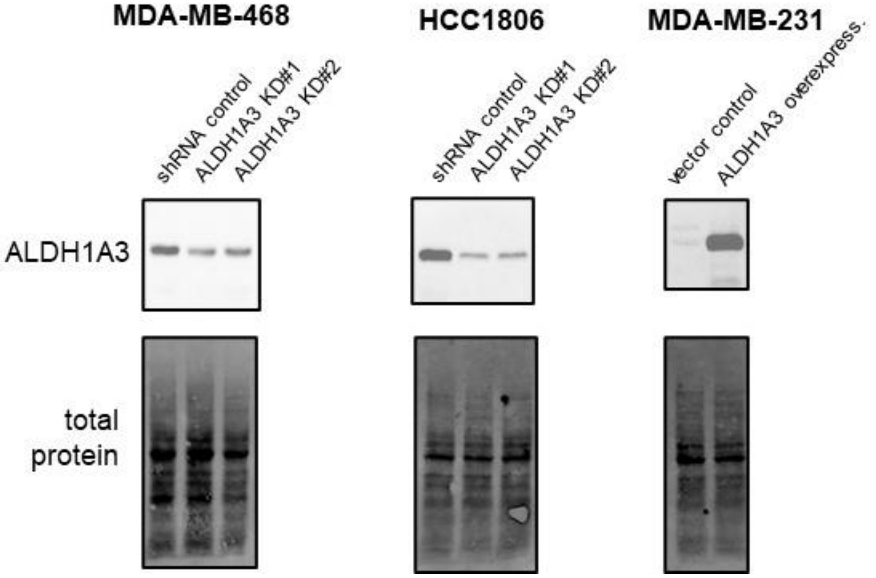

Supplemental Figure S1. Western blots confirm ALDH1A3 knockdown in MDA-MB-468 and HCC1806 cells and ALDH1A3 overexpression in MDA-MB-231 cells. The western blots also show that native ALDH1A3 levels are comparably high in MDA-MB-468 and HCC1806 cells and low in MDA-MB-231 cells.

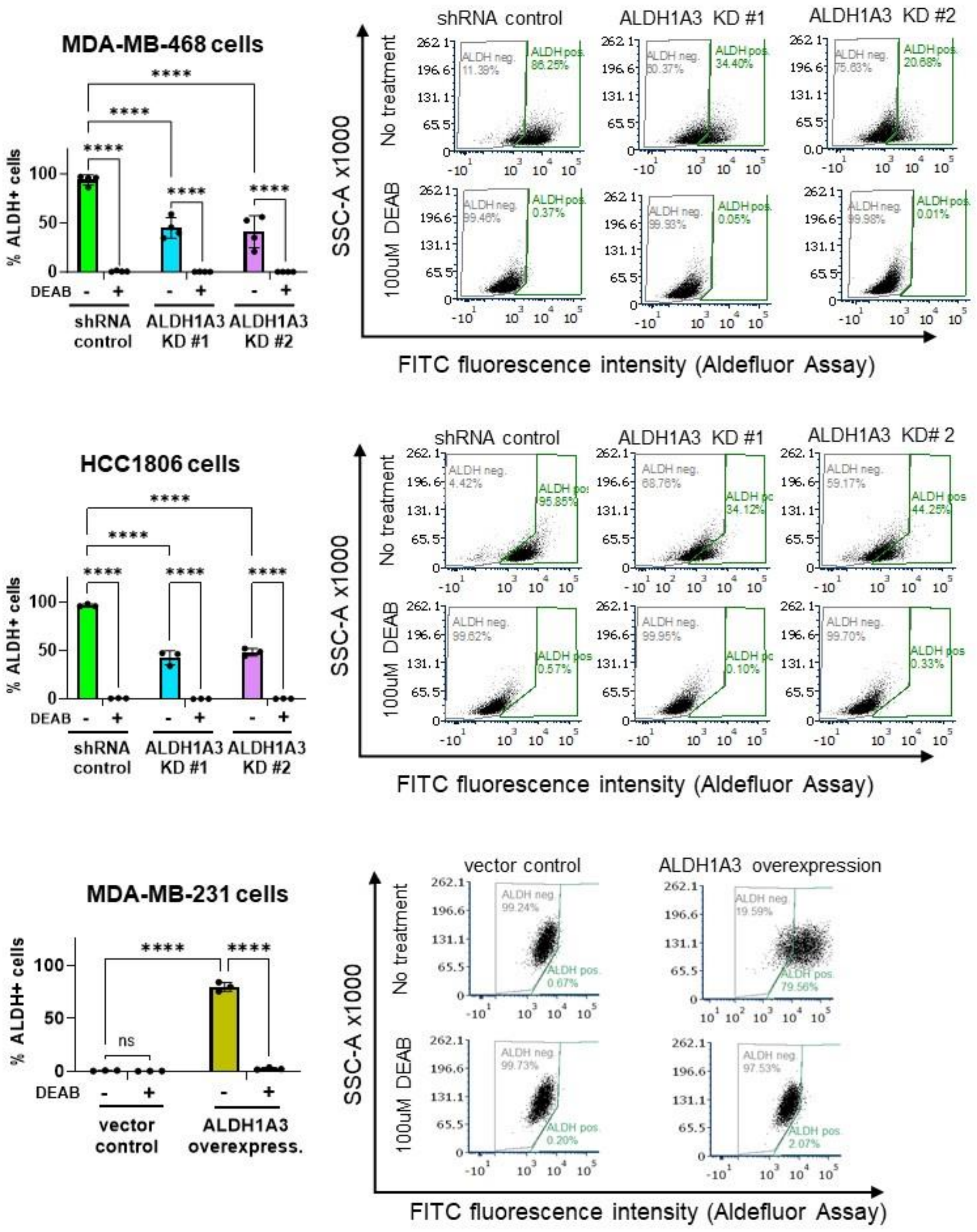

Supplemental Figure S2. The percentages of ALDH+ cells are decreased by ALDH1A3 knockdown in MDA-MB-468 and HCC1806 cells and increased by ALDH1A3 overexpression in MDA-MB-231 cells. The ALDH+ cells are determined by the Aldefluor assay and the addition DEAB serves as a control (pan-ALDH inhibitor) to determine the gating for ALDH- and ALDH+ cells. Significance determined by two-way Anova, followed by multiple comparison tests (3n, p value <0.0001=****, ns = not significant).

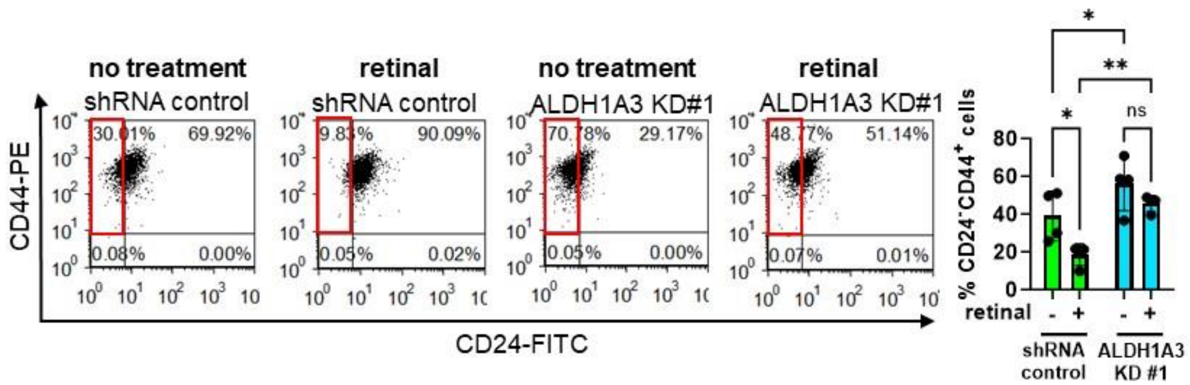

Supplemental Figure S3. Retinal reduction of CD24$^-$CD44$^+$ cells in HCC1806 cells is ALDH1A3 dependent. The effect of 24h 100nM retinal treatment in HCC1806 cells, with or without ALDH1A3 knockdown, on the percentage of CD24$^-$CD44$^+$ cells are determined by flow cytometry analysis of cell stained with anti-CD24-FITC conjugated and anti-CD44-PE conjugated antibody. The bar graph summarized the data of 4n. The error bars equal standard deviation and significance determined by two-way Anova, followed by multiple comparison post-tests (p value <0.05=*, <0.01=**, ns = not significant).

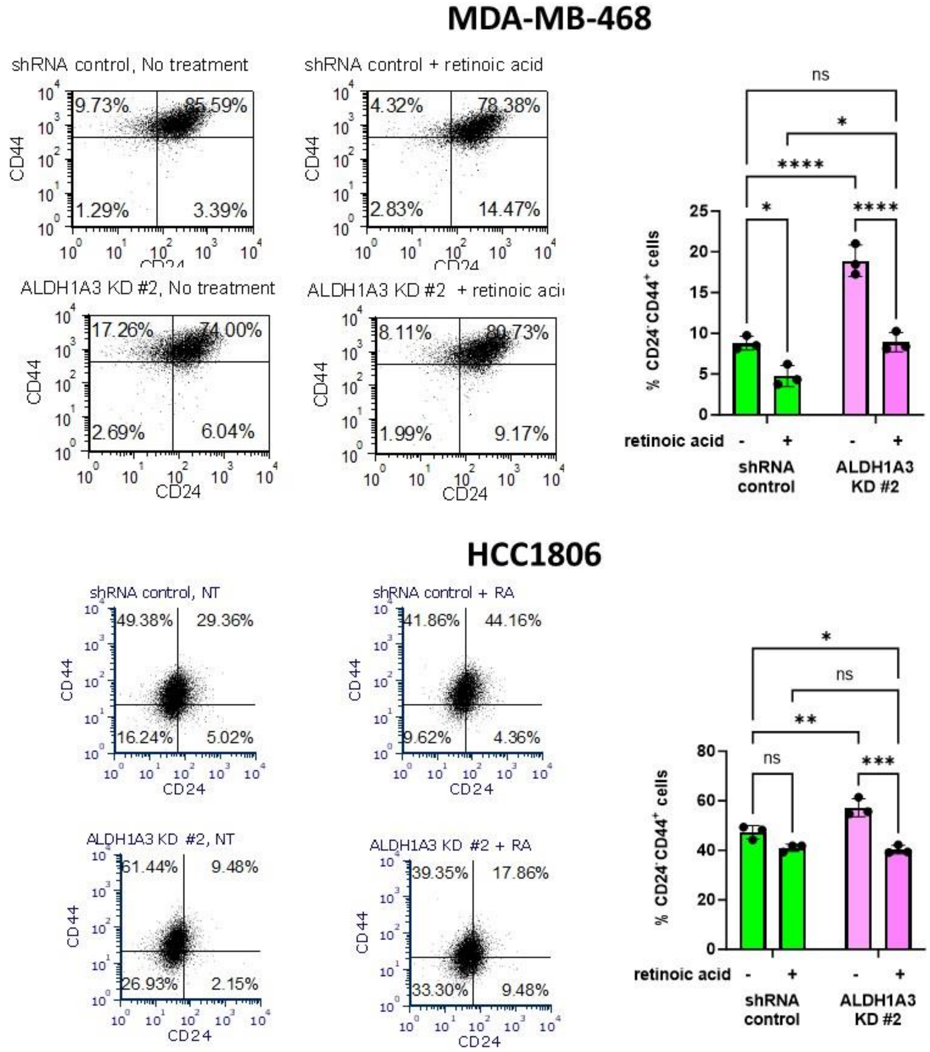

Supplemental Figure S4. Retinoic acid reduction of CD24$^-$CD44$^+$ cells in MDA-MB-468 and HCC1806 cells is independent of ALDH1A3. The effect of 24h 100nM retinoic acid treatment in MDA-MB-468 and HCC1806 cells, with or without ALDH1A3 knockdown, on the percentage of CD24$^-$CD44$^+$ cells is determined by flow cytometry analysis of cell stained with anti-CD24-APC conjugated and anti-CD44-PE conjugated antibody. The bar graph summarizes the data of 3n. The error bars equal standard deviation and significance determined by two-way Anova, followed by multiple comparison post-tests (p value <0.05=*, <0.01=**, ns = not significant).

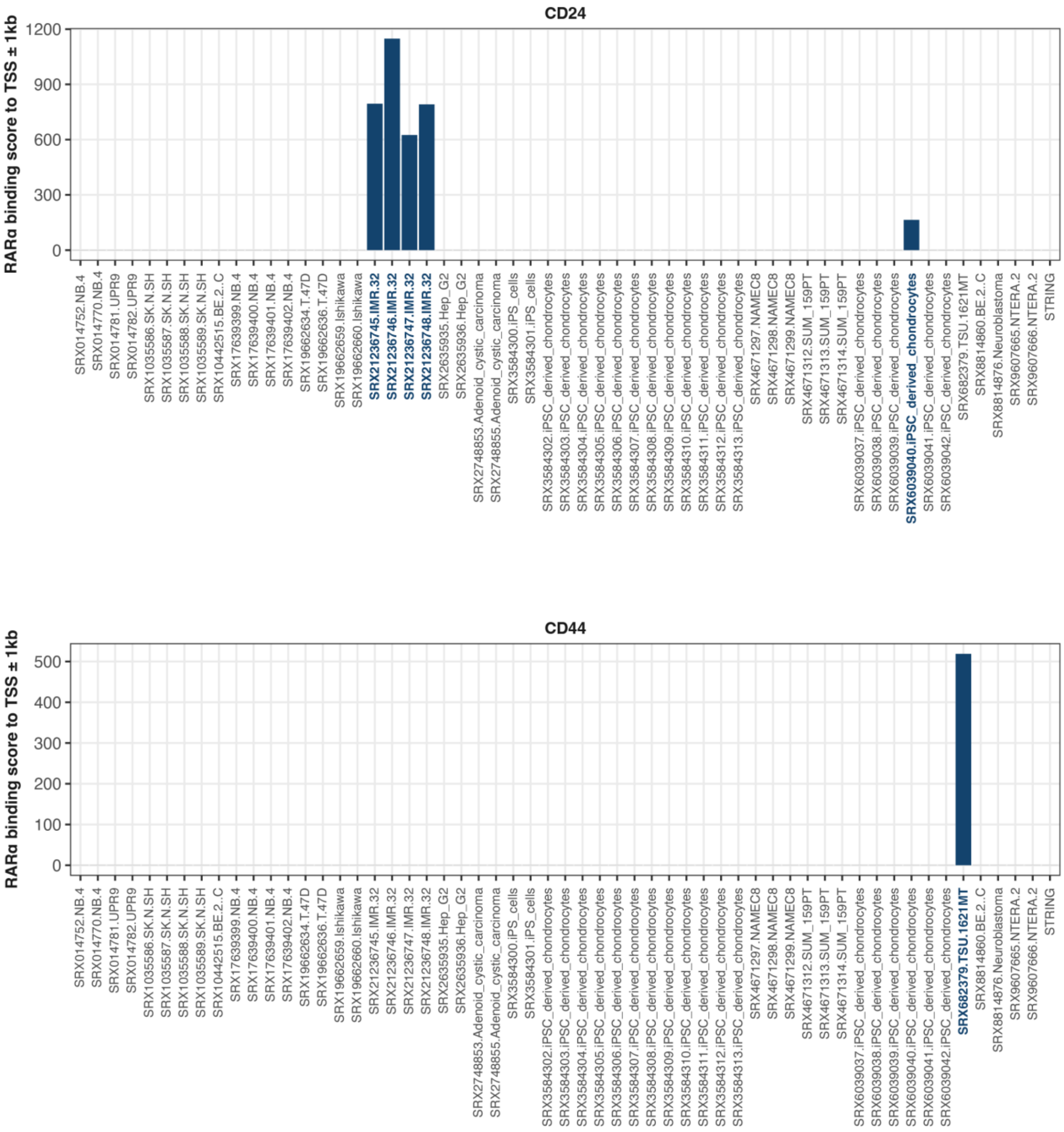

Supplemental Figure S5. RARA ChIPseq analysis reveals RARA binding within 1kb of transcription start site of CD24 in neuroblastoma IMR.32 cells and in IPSC derived chondrocytes and in CD44 in TSU-1621MT acute promylocytic leukemia cells. RARA binding scores for the cell line panel was extracted through the https://chip-atlas.org/target_genes site[3].

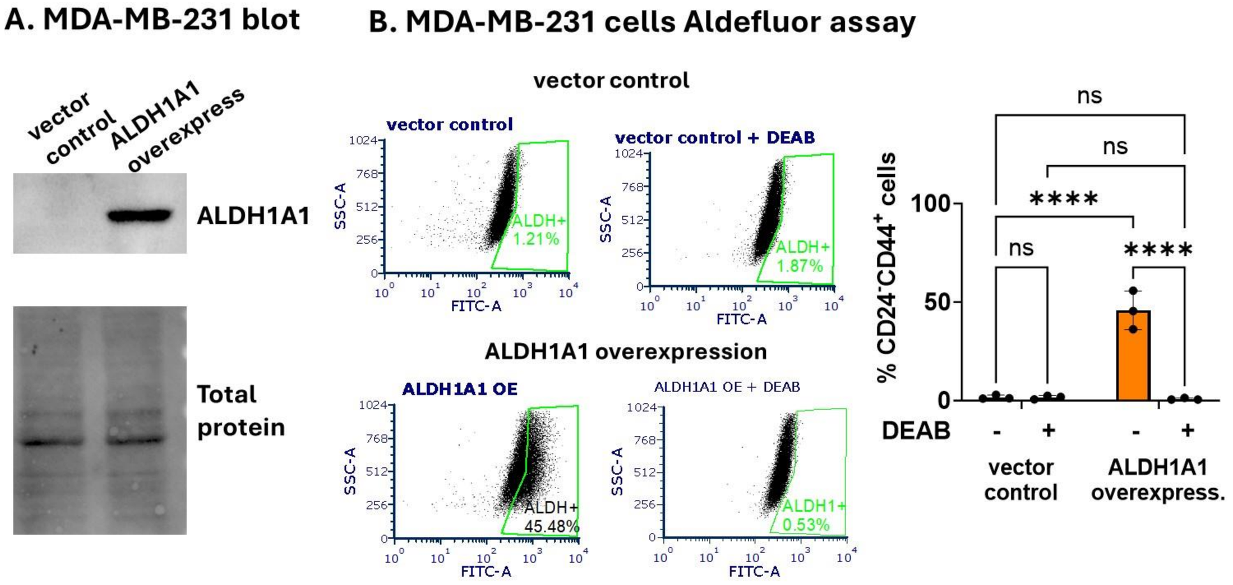

Supplemental Figure S6. Western blot confirms ALDH1A1 overexpression in MDA-MB-231 cells and Aldefluor assay shows increased ALDH$^+$ cells upon ALDH1A1 overexpression. A) The western blot shows the increased ALDH1A1 levels upon overexpression of ALDH1A1 in MDA-MB-231 cells. **B)** ALDH1A1 overexpression increases the percentage of positive ALDH+ cells as measured by the Aldefluor assay. Addition of 100uM DEAB (pan-ALDH inhibitor) inhibits Aldefluor activity. The bar graph is summary of 3n. The error bars equal standard deviation and significance determined by two-way Anova, followed by multiple comparison post-tests (p value <0.0001=****, ns = not significant).

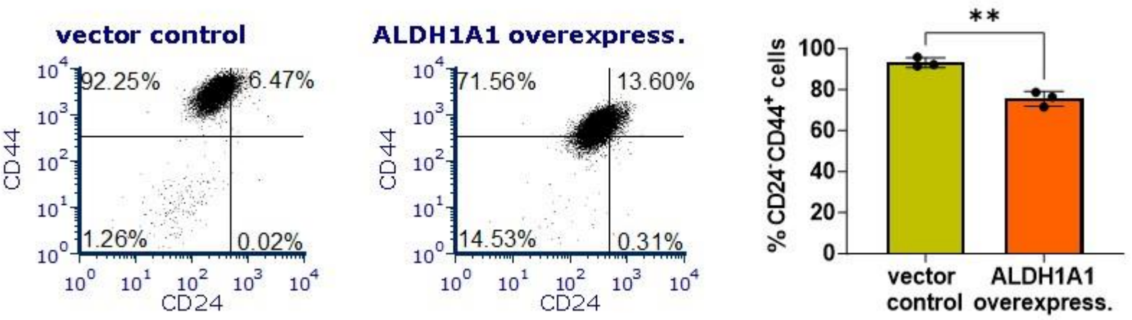

Supplemental Figure S7. ALDH1A1 overexpression reduces the CD24$^-$CD44$^+$ population in MDA-MB-231 cells. The effect of ALDH1A1 overexpression in MDA-MB-231 cells on the percentage of CD24$^-$CD44$^+$ cells is determined by flow cytometry analysis of cell stained with anti-CD24-APC conjugated and anti-CD44-PE conjugated antibody. The bar graph summarized the data of 3n. The error bars equal standard deviation and significance determined by two-way Anova, followed by multiple comparison post-tests (p value <0.01=**).

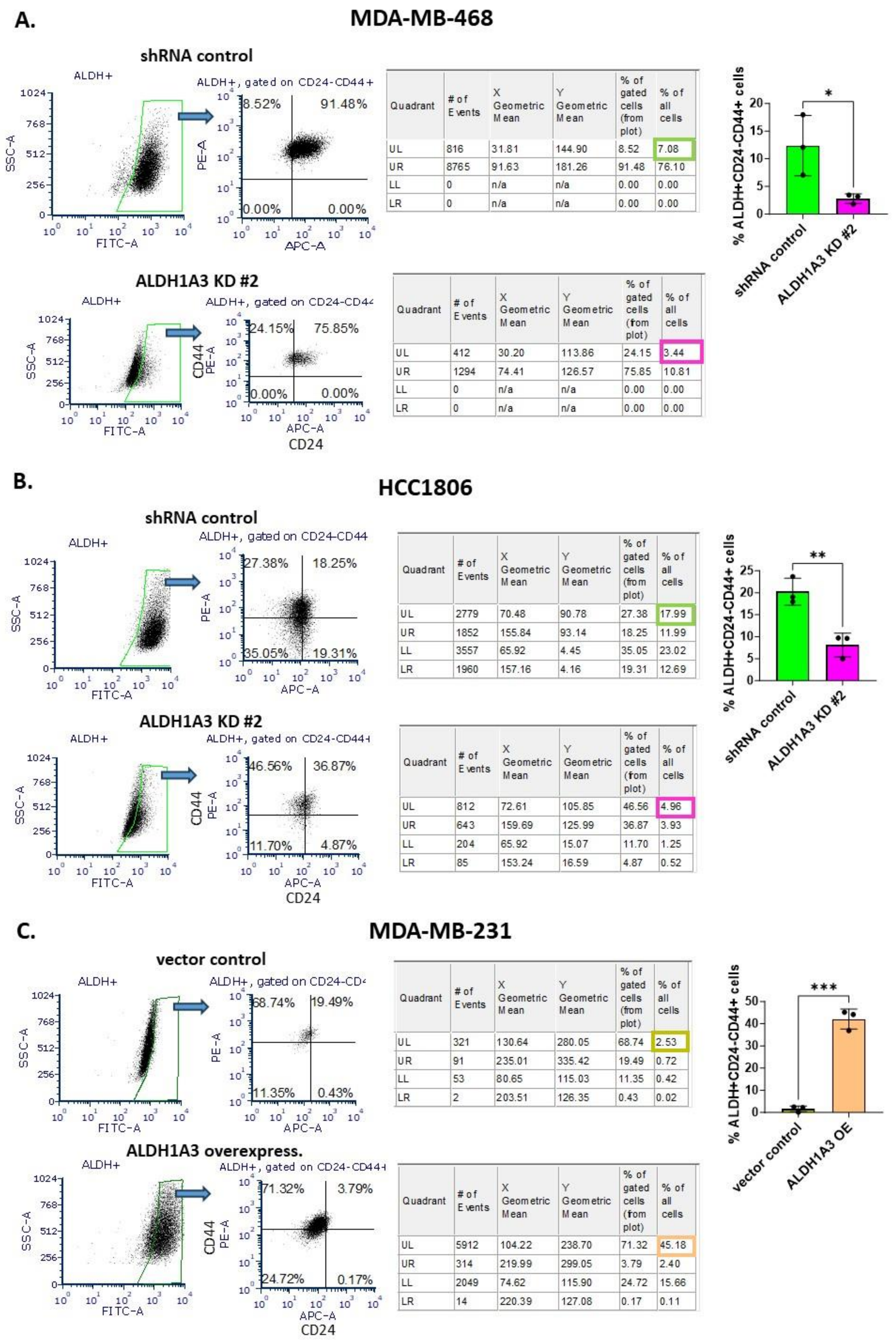

A. MDA-MB-468 — shRNA control

Quadrant	# of Events	X Geometric Mean	Y Geometric Mean	% of gated cells (from plot)	% of all cells
UL	816	31.81	144.90	8.52	7.08
UR	8765	91.63	181.26	91.48	76.10
LL	0	n/a	n/a	0.00	0.00
LR	0	n/a	n/a	0.00	0.00

A. MDA-MB-468 — ALDH1A3 KD #2

Quadrant	# of Events	X Geometric Mean	Y Geometric Mean	% of gated cells (from plot)	% of all cells
UL	412	30.20	113.86	24.15	3.44
UR	1294	74.41	126.57	75.85	10.81
LL	0	n/a	n/a	0.00	0.00
LR	0	n/a	n/a	0.00	0.00

B. HCC1806 — shRNA control

Quadrant	# of Events	X Geometric Mean	Y Geometric Mean	% of gated cells (from plot)	% of all cells
UL	2779	70.48	90.78	27.38	17.99
UR	1852	155.84	93.14	18.25	11.99
LL	3557	65.92	4.45	35.05	23.02
LR	1960	157.16	4.16	19.31	12.69

B. HCC1806 — ALDH1A3 KD #2

Quadrant	# of Events	X Geometric Mean	Y Geometric Mean	% of gated cells (from plot)	% of all cells
UL	812	72.61	105.85	46.56	4.96
UR	643	159.69	125.99	36.87	3.93
LL	204	65.92	15.07	11.70	1.25
LR	85	153.24	16.59	4.87	0.52

C. MDA-MB-231 — vector control

Quadrant	# of Events	X Geometric Mean	Y Geometric Mean	% of gated cells (from plot)	% of all cells
UL	321	130.64	280.05	68.74	2.53
UR	91	235.01	335.42	19.49	0.72
LL	53	80.65	115.03	11.35	0.42
LR	2	203.51	126.35	0.43	0.02

C. MDA-MB-231 — ALDH1A3 overexpress.

Quadrant	# of Events	X Geometric Mean	Y Geometric Mean	% of gated cells (from plot)	% of all cells
UL	5912	104.22	238.70	71.32	45.18
UR	314	219.99	299.05	3.79	2.40
LL	2049	74.62	115.90	24.72	15.66
LR	14	220.39	127.08	0.17	0.11

Supplemental Figure S8. ALDH1A3 increases the percentage of ALDH$^+$CD24$^-$CD44$^+$ population in TNBC cell lines. The Aldefluor assay was completed on MDA-MB-468 **(A),** HCC1806 **(B)** or MDA-MB-231 cells **(C)** in the presence of anti-CD24-APC conjugated and anti-CD44-PE conjugated antibodies. ALDH$^+$ cells were gated on based on the addition of DEAB pan-ALDH inhibitor. The ALDH$^+$ cells were gated for assessment of CD24$^-$CD44$^+$ cells, and the percentage of ALDH$^+$CD24$^-$CD44$^+$ cells was determined from all of the cells (3n). Significance is determined by t-test and compared shRNA control to ALDH1A3 knockdown (**A, B**) or vector control to ALDH1A3 overexpression (**C**). P value <0.05=*, <0.01=**, <0.001=***.

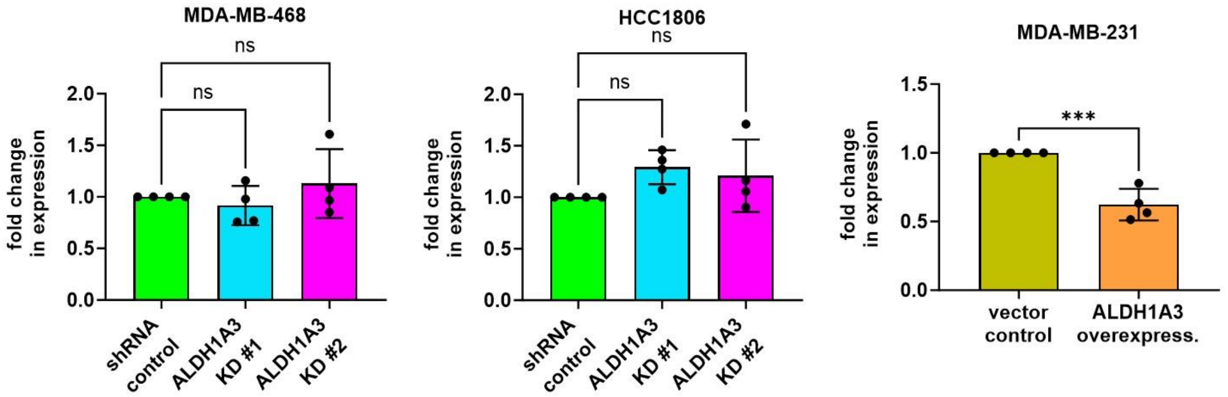

Supplemental Figure S9. ITGB4 mRNA expression is reduced by ALDH1A3 overexpression in MDA-MB-231 cells. The effect of ALDH1A3 knockdown or overexpression on the relative mRNA transcript levels of integrin beta 4 (ITBB4) is determined by quantitative polymerase chain reaction (RT-qPCR), relative to two reference genes and the control in MDA-MB-468, HCC1806, and MDA-MB-231 cells. Significance was determined by one-way Anova followed by multiple comparison post-tests for MDA-MB-468 and HCC1906 cells or t-test for MDA-MB-231 cells. P value <0.001=***, ns= not significant. Error bars represent standard deviation.

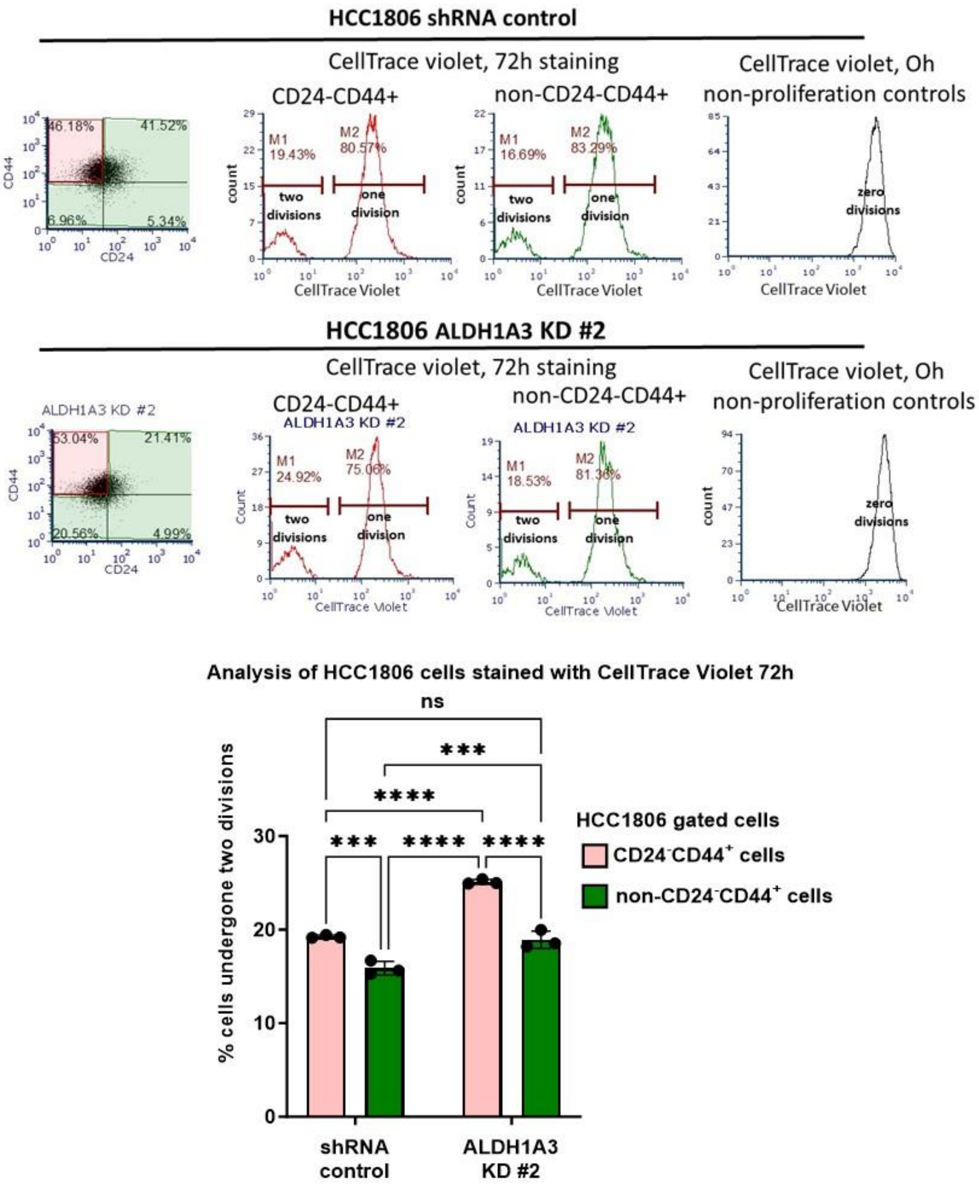

Supplemental Figure S10. ALDH1A3 knockdown and CD24$^-$CD44$^+$ cells in HCC1806 cells have increased cell proliferation. The HCC1806 shRNA control and ALDH1A3 KD #2 cells were stained with CellTrace Violet and cultured for 72h before analysis before staining with CD24-APC and CD44-PE conjugated antibodies by flow cytometry. Cells were gated based on CD24 and CD44 expression (CD24$^-$CD44$^+$ versus non-CD24$^-$CD44$^+$). Cells that had divided one or twice were identified by the reduction in CellTrace violent staining. Cell stained with CellTrace Violet witthout culturing served as a zero division non-prolilferation control. The percentages from of cells that had gone the most divisions (lowest CellTrace Violet) were ploted in the bargraphs. The error bars equal standard deviation and significance determined by two-way Anova, followed by post-tests (p value <0.001=***, <0.0001=****, ns = not significant).

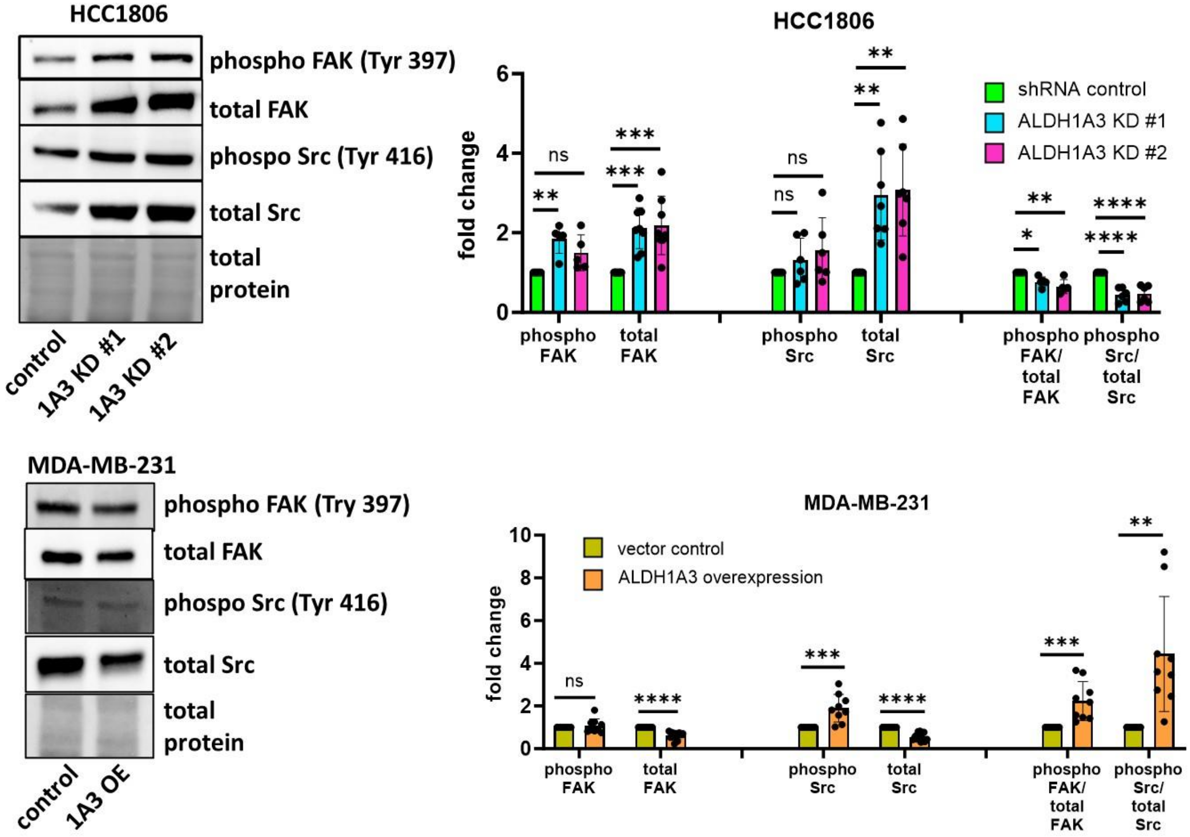

Supplemental Figure S11. Western blots of HCC1806 and MDA-MB-2321 cells show ALDH1A3 decreases total FAK and Src, but not phosphorylation of the proteins, leading to increased phospho FAK/total FAK and phospho Src/total Src ratios. The blots on the left are representative replicates and the bar graphs summarize the image band quantification of individual biological replicates relative to the total protein and the control and the ratio of phosphorylated FAK or Src versus total FAK or Src. Significance determined by one-way Anova followed by multiple comparison post-tests for MDA-MB-468 and HCC11806 cells and by t-test for MDA-MB-231 cells. P value <0.05=*, <0.01=**, <0.001=***, <0.0001=****, ns=not significant. Error bars represent standard deviation.

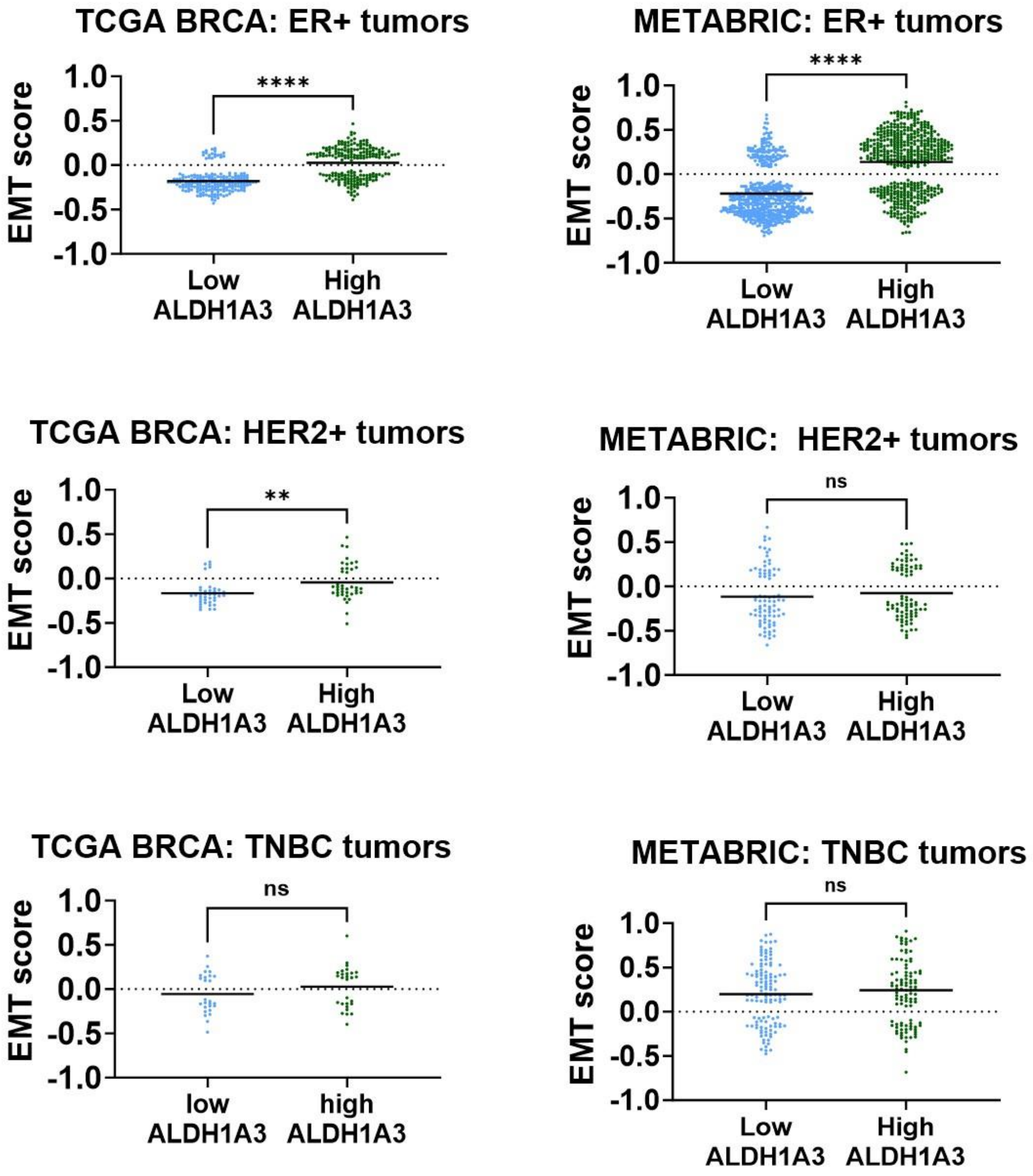

Supplemental Figure S12. EMT Score of breast cancer patient tumor clinical subtypes grouped based on low versus high ALDH1A3 expression. The EMT score is calculated for individual patient tumors using expression data available for TCGA BRCA (Cell 2015) and METABRIC datasets identified as ER+, HER2+ or TNBC. Patient tumors are grouped as either low or high ALDH1A3 based on ranking of being in the bottom third for ALDH1A expression or top third of all breast cancer patients within each subset in the dataset. Significance determined t-test (p value <0.01=**, <0.0001=****, ns = not significant).

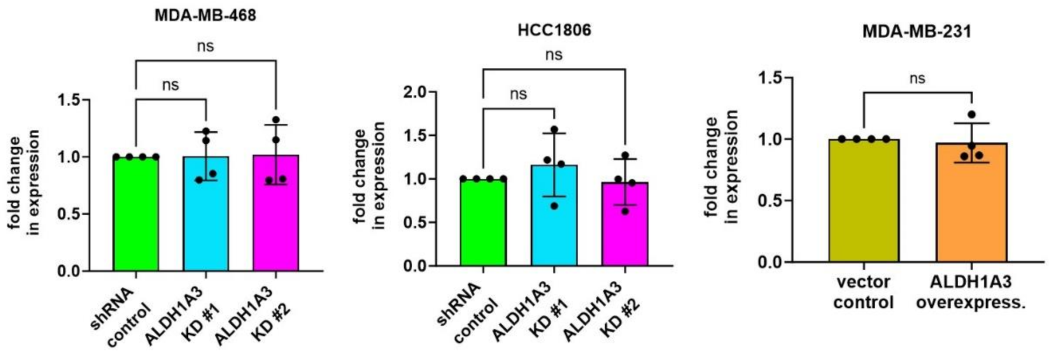

Supplemental Figure S13. NRF2 mRNA expression is not altered by ALDH1A3 in MDA-MB-468, HCC1806, and MDA-MB-231 cells. The effect of ALDH1A3 knockdown or overexpression on the relative mRNA transcript levels of nuclear factor erythroid 2-related factor 2 (NRF2), also called nuclear factor erythroid-derived 2-like 2 (NFE2L2) is determined by quantitative polymerase chain reaction (RT-qPCR), relative to two reference genes and the control in MDA-MB-468, HCC1806, and MDA-MB-231 cells. Significance was determined by one-way Anova followed by multiple comparison post-tests for MDA-MB-468 and HCC1906 cells or t-test for MDA-MB-231 cells. Error bars represent standard deviation.

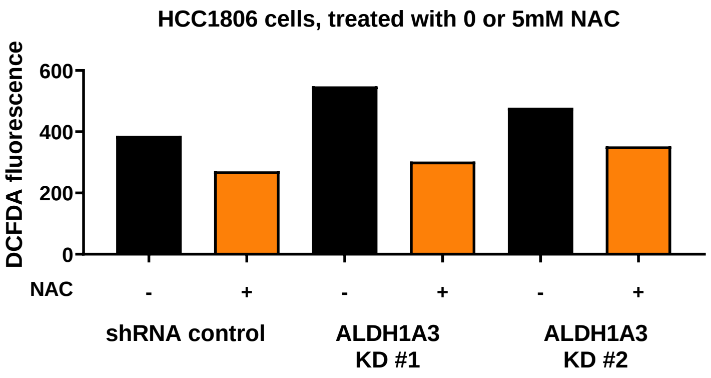

Supplemental Figure S14. Reactive oxygen scavenger N-acetylcysteine (NAC) reduces increased DCFDA fluorescence associated with ALDH1A3 knockdown in HCC1806 cells. Cells seeded in 6 well plates were treated with 0 or 5mM NAC for 24 h and then stained with DCFDA prior to flow cytometry analysis (n=1).

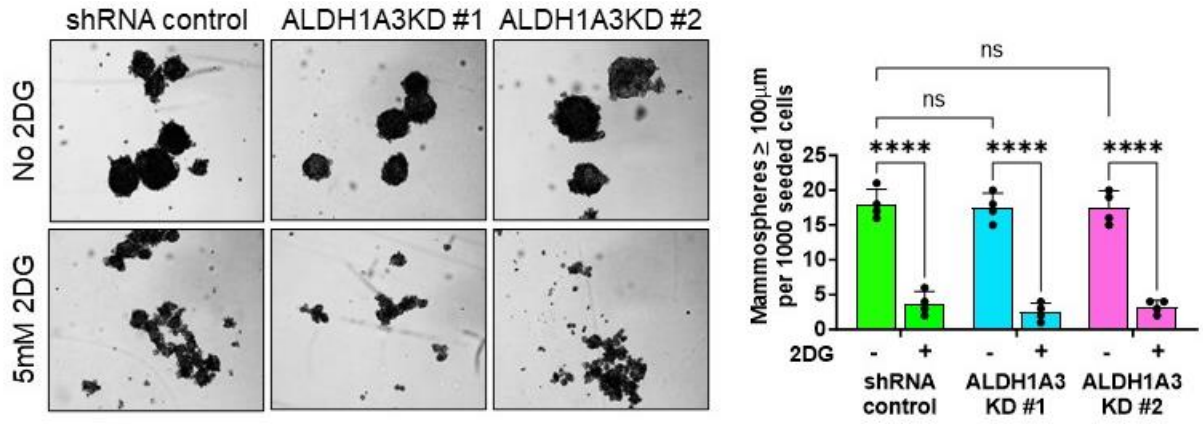

Supplemental Figure S15. 2DG treatment reduces mammosphere formation in HCC1806 cells. The control and ALDH1A3 knockdown cells were treated with 5mM 2DG for 48 h and then 1000 cells were washed and re-cultured in mammosphere media in 24 well ultra-low adherent plates and 5 days later the resulting cultures were imaged and spheroids larger than 100µm counted (bar graph). The error bars equal standard deviation and significance determined by two-way Anova, followed by multiple comparison post-tests (p value <0.0001=****, ns = not significant).

Supplemental Table S1: Sequences of the primers used for this study.

Name of the gene	Primer sequence	
	Forward	Reverse
ARF1	GTGTTCGCCAACAAGCAGG	CAGTTCCTGTGGCGTAGTG
ATP6V0A4	CTGGCTCATCAAGGTGCAGA	CAGATCTCGGCGATGACACA
B2M	AGGCTATCCAGCGTACTCCA	CGGATGGATGAAACCCAGACA
CD24	GCTCCTACCCACGCAGATTT	GAGACCACGAAGAGACTGGC
CD44	CCCATTCGACAACAGGGACA	AGCTGAGGTCACTGGGATGA
CDH1	GGAGAGCGGTGGTCAAAGAG	AGTCCTGGTCCTCTTCTCCG
CDH2	CCACTGCTGGGTCCTTGAG	CCCCCAGTCGTTCAGGTAATC
CLDN1	TTGGGCTTCATTCTCGCCTT	GTCGCCGGCATAGGAGTAAA
CLDN8	GTGGATGAATTGCGTGAGGC	TAGGTCCGGAGAAAGAGCCA
ENO1	CCTTCATCGCTGACCTGGTT	AGGAGCTGGTTGTACTTGGC
ENO2	GGATGTTGCTGCCTCAGAGT	ATCGGGAAGGATCAGTGGGA
ITGB4	AGAGTCCCAGGATGGAGGAC	GGTGGTGATGCTGCTGTACT
MMP2	GGCGGTCACAGCTACTTCTT	GCCTAGCCAGTCGGATTTGA
NRF2	AGGTTGCCCACATTCCCAAA	AATGTCTGCGCCAAAAGCTG
OCLN	TCGACCAATGCTCTCTCAGC	CTCCTGGAGGAGAGGTCCAT
PUM1	GGCGTTAGCATGGTGGAGTA	CATCCCTTGGGCCAAATCCT
SNAI2	CCAAGCTTTCAGACCCCCAT	TGCAGCTGCTTATGTTTGGC
TBP	GGCACCACTCCACTGTATCC	GCTGCGGTACAATCCCAGAA
TPI	AGCAGACAAAGGTCATCGCA	TGTTGGGGTGTTGCAGTCTT
TWIST1	CTCGGACAAGCTGAGCAAGA	GCTCTGGAGGACCTGGTAGA
VIM	AAATGGCTCGTCACCTTCGT	CAGCTTCCTGTAGGTGGCAA

Supplemental Table S2: Limiting dilution assay data for MDA-MB-468 ALDH1A3 knockdown and 2DG treated tumors from Fig. 7.

	**shRNA control, no treatment, # of mice that developed tumors**		
	tumor harvested from mouse #1	tumor harvested from mouse #2	tumor harvested from mouse #3
number of cells injected	number of mice developed new tumors	number of mice developed new tumors	number of mice developed new tumors
5000	1/4	0/4	1/4
50000	0/4	2/4	2/4
500000	2/4	2/2	2/4
**ELDA, TIC frequency estimate**	**1/547480**	**1/81380**	**1/307232**

	**shRNA control tumors, treated with 2DG, # of mice that developed new tumors**		
	tumor harvested from mouse #1	tumor harvested from mouse #2	
number of cells injected	number of mice developed new tumors	number of mice developed tumors	
5000	3/4	1/4	
50000	3/4	1/4	
500000	1/4	1/2	
**ELDA, TIC frequency estimate**	**1/258248**	**1/291172**	

**ALDH1A3 KD #1 tumors, no treatment, # of mice that developed new tumors**			
	tumor harvested from mouse #1	tumor harvested from mouse #2	tumor harvested from mouse #3
number of cells injected	number of mice developed new tumors	number of mice developed new tumors	number of mice developed new tumors
5000	1/4	2/4	4/4
50000	1/4	4/4	3/4
500000	4/4	3/4	3/4
**ELDA, TIC frequency estimate**	**1/90978**	**1/99453**	**1/88925**

**ALDH1A3 KD #1 tumors, treated with 2DG, # of mice that developed new tumors**			
	tumor harvested from mouse #1	tumor harvested from mouse #2	tumor harvested from mouse #3
number of cells injected	number of mice developed tumors	number of mice developed tumors	number of mice developed tumors
5000	3/4	0/4	2/4
50000	2/4	1/2	0/4
500000	1/4	2/4	4/4
**ELDA, TIC frequency estimate**	**1/307704**	**1/504434**	**1/100517**

Mice in each group were scorered for tumor development. The tumor initiating cell (TIC) frequency for each tumor was estimated from this data using the extreme limiting dilution method (https://bioinf.wehi.edu.au/software/elda/), Hu, Y, and Smyth, GK (2009). ELDA: Extreme limiting dilution analysis for comparing depleted and enriched populations in stem cell and other assays. *Journal of Immunological Methods* 347, 70-78.

Supplemental Table S3: Limiting dilution assay data for HCC1806 knockdown and 2DG treated tumors from Fig. 7.

HCC1806 limiting dilution assay, mice scored for new tumor development			
	shRNA control, no treatment, # of mice that developed tumors		
	tumor harvested from mouse #1	tumor harvested from mouse #2	tumor harvested from mouse #3
number of cells injected	number of mice developed new tumors	number of mice developed new tumors	number of mice developed new tumors
10	2/6	1/6	1/6
100	2/4	2/6	2/6
1000	4/6	1/2	4/6
**ELDA, TIC frequency estimate**	**1/459**	**1/472**	**1/571**
	shRNA control tumors, treated with 2DG, # of mice that developed new tumors		
	tumor harvested from mouse #1	tumor harvested from mouse #2	tumor harvested from mouse #3
number of cells injected	number of mice developed new tumors	number of mice developed tumors	number of mice developed tumors
10	3/6	1/6	1/6
100	4/6	2/6	3/6
1000	2/6	2/6	3/6
**ELDA, TIC frequency estimate**	**1/574**	**1/1080**	**1/662**
ALDH1A3 KD #1 tumors, no treatment, # of mice that developed new tumors			
	tumor harvested from mouse #1	tumor harvested from mouse #2	tumor harvested from mouse #3
number of cells injected	number of mice developed new tumors	number of mice developed new tumors	number of mice developed new tumors
10	3/6	1/2	2/4
100	4/6	6/6	1/4
1000	3/6	3/6	3/6
**ELDA, TIC frequency estimate**	**1/441**	**1/426**	**1/760**
ALDH1A3 KD #1 tumors, treated with 2DG, # of mice that developed new tumors			
	tumor harvested from mouse #1	tumor harvested from mouse #2	tumor harvested from mouse #3
number of cells injected	number of mice developed tumors	number of mice developed tumors	number of mice developed tumors
10	2/6	1/4	2/6
100	3/6	1/6	2/6
1000	2/6	1/4	1/6
**ELDA, TIC frequency estimate**	**1/775**	**1/1341**	**1/1196**

Mice in each group were scorered for tumor development**.** The tumor initiating cell (TIC) frequency for each tumor was estimated from this data using the extreme limiting dilution method (https://bioinf.wehi.edu.au/software/elda/), Hu, Y, and Smyth, GK (2009). ELDA: Extreme limiting dilution analysis for comparing depleted and enriched populations in stem cell and other assays. *Journal of Immunological Methods* 347, 70-78.
